# Supplementary material for: Predatory synapsid ecomorphology signals growing dynamism of late Palaeozoic terrestrial ecosystems
Source: Commun Biol. 2024 Feb 17;7:201. doi: 10.1038/s42003-024-05879-2 (PMC10874460; doi:10.1038/s42003-024-05879-2)
Supplement: Supplementary file 2 — Supplementary Information [file 42003_2024_5879_MOESM2_ESM.pdf]

Predatory synapsid ecomorphology signals growing dynamism of late Palaeozoic terrestrial ecosystems.

Suresh A. Singh[1*], Armin Elsler[1], Thomas L. Stubbs[2], Emily J. Rayfield[1], and Michael J. Benton[1].

[1]School of Earth Sciences, University of Bristol, Life Sciences Building, Tyndall Avenue, Bristol, BS8 1TQ, United Kingdom.
[2]School of Life, Health & Chemical Sciences, Open University, Milton Keynes, MK7 6AE, United Kingdom
*Corresponding Author: sureshsingh.palaeo@gmail.com

Supplementary Materials:

Contents:

Supplementary Methods……………………………………………………………5
Mandibular functional characters

Supplementary Methods……………………………………………………………7
Size data

Supplementary Methods……………………………………………………………7
Phylogenetic tree

Supplementary Table 1……………………………………………………………8
Shape principal component analysis eigenvalues

Supplementary Table 2……………………………………………………………12
Functional principal component analysis eigenvalues

Supplementary Table 3……………………………………………………………13
Functional principal component analysis character loadings.

Supplementary Table 4……………………………………………………………14
PERMANOVA results for shape differences between synapsid carnivore clades.

Supplementary Table 5……………………………………………………………15

PERMANOVA results for functional differences between synapsid carnivore clades.

Supplementary Table 6…..……………………………………..……………..…..16
Internal validation statistics for cluster configurations.

Supplementary Table 7.……………………………………..………..………..17
External validation statistics for different cluster configurations.

Supplementary Table 8……………………………………..…………….....……....18
PERMANOVA results for significant differences between synapsid carnivore mandibular shape and function between feeding functional groups.

Supplementary Table 9.……………………………………..………………....19
PERMANOVA results for significant differences in synapsid carnivore mandibular shape between feeding functional subgroups.

Supplementary Table 10..……………………………………………………...…20
PERMANOVA results for significant differences in synapsid carnivore mandibular function between feeding functional subgroups.

Supplementary Table 11..……………………………..…………………..….……..21
Support for different macroevolutionary models of mandibular functional disparity evolution.

Supplementary Table 12..……………………………………………...……….…....22
PERMANOVA results for significant differences between synapsid carnivore mandibular shape and function between geological stages.

Supplementary Table 13...…………………..……………………………..…...…....23
Mann-Whitney U test results for significant body size changes in synapsid carnivores between geological stages.

Supplementary Table 14...……………………………..………………..…..……..23
Support for different models of ancestral trait estimation of feeding functional subgroup.

Supplementary Figure 1………………………………………………………….…...….24
Geometric morphometric landmarking regime.

Supplementary Figure 2……………………………………………………...……..25
Functional character measurements guide.

Supplementary Figure 3……………………………………………………….......26
Secondary carnivorous synapsid mandibular morphofunctional morphospaces.

Supplementary Figure 4………………………………………………………………27
Carnivorous synapsid mandibular functional character distributions.

Supplementary Figure 5………………………………………………………………29
Carnivorous synapsid feeding functional group mandibular characteristics.

Supplementary Figure 6…………………………………………………......…..31
Carnivorous synapsid feeding functional group validation.

Supplementary Figure 7………………………………………………………………32
Carnivorous synapsid size distributions across taxonomic and feeding functional (sub)groups.

Supplementary Figure 8…………………………………………………………..…...33
Carnivorous synapsid size distributions by taxonomic and feeding functional group through the late Palaeozoic.

Supplementary Figure 9………………………………………………………...…..34
Equal rates ancestral trait reconstruction of carnivorous synapsid FFsG through the late Palaeozoic.

Supplementary Figure 10……………………………………….……………...…...35
Symmetrical rates ancestral trait reconstruction of carnivorous synapsid FFsG through the late Palaeozoic.

Supplementary Figure 11………………………………………………………...…...36

Asymmetrical rates ancestral trait reconstruction of carnivorous synapsid FFsG through the late Palaeozoic.

Supplementary Figure 12…………………………………………………………..…...…..37
All rates different ancestral trait reconstruction of carnivorous synapsid FFsG through the late Palaeozoic.

Supplementary Note 1….…...…………....…………………………………...……….38
R Code

References…………………….…………….…….……………………………….....42

Supplementary Methods

Mandibular Functional Characters. The functional characters used here are taken from the study by Singh et al.[1] of mandibular function across a wide range of early Mesozoic taxa. See Supplementary Fig. 2 for guidance on how each character is measured.

Characters 1–3 are based on using lever mechanics to describe mandibular function, with the jaw acting as a third-order lever system[2,3]. The adductor musculature provides the input force, the craniomandibular joint acts as a fulcrum, and an output force is produced along the toothrow. Levers are measured from the craniomandibular joint, which is the point of overall jaw articulation, to a point along the jaw margin that is perpendicular to the mid-point of the relevant muscle (Fig. 1). Using margins points provides distinct points that avoid the uncertainty that may arise from trying to identify the exact centre of muscle group attachments across specimens. Taxa with low mechanical advantage (MA) typically exhibit weak, fast bites[4,5]. Inversely, taxa with high MA possess slower, more powerful bites. In characters one and two, the distance from the jaw adductor muscle attachment to the jaw joint represents the inlever. For characters one and two, we use the mean MA values generated from the MA of each of the three main jaw muscle groups as a more cautious measure of MA, although it should be noted that this inherently reduces the signal of therapsid jaw musculature modifications[6]. These modifications also encompass the earliest stages of jaw anatomy reorganisation that eventually produced the novel mammalian jaw and ear[7-10].

Reorganisation of the adductor musculature and wider jaw anatomy say shifts in the attachment of the adductor muscles on the lower jaw, resulting in slightly different positions for the inlever tangents required to calculate MA across basal synapsids, non-cynodont therapsids and cynodont therapsids (Fig. 1). For basal synapsids, we largely follow the pattern of adductor muscle attachment laid out by Barghusen[11], in which the attachments of the primary adductor muscle groups (adductor mandibulae externus – MAME, posterior – MAMP, and internus pterygoideus - MAMIPt) are not especially derived from that of sauropsids. Therefore, in more basal groups such as varanopids, we defer to attachment reconstructions as used for other sauropsid groups[8,12,13]. The MAME and MAMP attach to the dorsal, dorsolateral, and dorsomedial margins of the lower jaw, between the coronoid and jaw articulation, while the MAMIPt attached further posteroventrally, to the lower margin of the angular, immediately posterior to the reflected lamina[11] (Fig. 1). Jaw modification across the basal synapsid-therapsid transition changes the angle and length of the moment arms[6-8,10], but the muscle group attachment sites on the lower jaw generally remain quite similar to as seen in *Dimetrodon*[11,14]. The expanded reflected lamina present in therapsids required careful consideration of the literature and both the lateral and medial view images of the jaw where available to assess angular morphology and avoid reconstructing muscle insertions on the reflected lamina, although gorgonopsians are

thought to have had some attachment on this structure[15,16]. Thrinaxodontid cynodonts also required a slightly different regime as the adductor musculature attachment began to shift from the postdentary bones onto the dentary in these early cynodonts[9,14,17].

1. Mean Anterior Mechanical Advantage: Biting efficiency at the anterior of the mandible, which marks the furthest bite point from the craniomandibular joint, producing the lowest possible MA. This ratio of the inlever to the outlever uses the distance from the jaw joint to the anterior-most tip of the toothrow/dentary as the length of the outlever.
2. Mean Posterior Mechanical Advantage: Biting efficiency at the posterior of the mandible, which marks the highest possible value of MA. This ratio of the inlever to the outlever uses the distance from the jaw joint to the posterior-most tip of the toothrow/dentary as the length of the outlever.
3. Opening Mechanical Advantage: This measure assesses biting velocity[2]. It is the ratio of the maximum inlever to the maximum outlever, using the distance from the jaw joint to the posterior-most point of the mandible/retroarticular process for the inlever, and using the distance from the jaw joint to the posterior-most point of the toothrow/dentary as the outlever. Opening MA is linked to feeding patterns and prey selection[5,18].
4. Relative Maximum Aspect Ratio: A 2D proxy for the second moment of area[1,5,19]. Generated by dividing the maximum depth of the mandible by its total length. Most mandibles primarily experience dorsoventral stress during feeding function, therefore the aspect ratio captures flexural stiffness[20]. Higher values may reflect greater stresses involved in carnivorous synapsid feeding behaviour.
5. Relative Toothrow Length: A measure of the relative length of the toothrow and its supposed importance in feeding behaviour[21]. Generated by dividing the length of the toothrow by the total length of the mandible. A longer toothrow enables a greater range of MA along the jaw and likely supported gripping ability in carnivorous synapsids.
6. Relative Symphysis Length: Represents symphyseal robusticity and is calculated by dividing the length of the symphysis by the total length of the mandible. The symphysis is subject to significant bending, shearing, and torsional stresses during biting action, and is also closely related to the transmission of biting forces, making it an important part of the jaw and key to understanding feeding ecology and overall jaw mechanics[22,23]. High values may suggest greater stresses involved in synapsid carnivore feeding behaviour.
7. The symphyseal angle is measured between the ventral jaw line and a line parallel to the long axis of the mandibular symphysis. This angle affects symphyseal resistance to bending, shear, and torsional stresses during the bite cycle[22]. Variation in the symphyseal angle of modern crocodylians has been found to relate to their mechanical response to biting, twisting, and shaking[24,25], making this character informative for inferring prey capture behaviours in carnivorous synapsids.

8. Relative Offset of Articulation: This character is measured as the length of the line perpendicular to the tangent of the mandibular toothrow (extrapolated from the anterior and posterior-most points of the toothrow to account for jaw curvature) which intersects the articular joint[20,21]. This value is then divided by the total length of the mandible. The offset between the toothrow and jaw joint affects dental occlusion and the leverage of the jaw musculature[26]. A low articulation offset value indicates 'scissor-like' occlusion, which is typical of many carnivorous taxa. A larger offset allows for better occlusion across the entire toothrow and is typically associated with gripping & crushing functions[27].

Size Data. In this study, we used basal skull length, defined here as the distance between the posterior most point on the lateral articular condyle of the quadrate to the ventrorostral tip of the premaxilla (vertically projected distance). In taxa with teeth, the rostral end of the distance corresponds to the rostral end of the alveolus of the first pair of teeth. Additionally, where possible we also recorded the sagittal/midline skull length, which we define the anterior most point on premaxilla to a line connecting the posterior points on the occipital condyles visible in ventral view, which is also used as basal skull length in some studies[28].

Phylogeny. The overall tree topology was based on Mann and Paterson[29], which further expanded and adapted the character matrices used by Benson[30], Reisz and Fröbisch[31], and Brocklehurst et al.[32]. Scaffolds for major subclades are based on Brink et al.[33] (early Therapsida), Liu[34] (Dinocephalia), Bendel et al.[35] (Gorgonopsia), Liu and Abdala[36] (Therocephalia), Abdala et al.[37] (early Cynodontia).

Supplementary Tables.

Supplementary Table 1. Shape (geometric morphometric data) principal component analysis eigenvalues. Abbreviation: PC, principal component.

Principal Component	Eigenvalues	Proportion Of Variation	Cumulative Variation
PC1	0.002302956	0.277837661	0.277837661
PC2	0.001072666	0.129410697	0.407248358
PC3	0.000775706	0.093584232	0.50083259
PC4	0.000617804	0.07453434	0.57536693
PC5	0.000452951	0.054645784	0.630012714
PC6	0.000404658	0.048819557	0.678832271
PC7	0.000294625	0.035544691	0.714376962
PC8	0.000252521	0.030465066	0.744842027
PC9	0.000169029	0.020392361	0.765234389
PC10	0.000160796	0.019399026	0.784633415
PC11	0.00013746	0.016583724	0.801217139
PC12	0.000121542	0.014663342	0.815880481
PC13	0.00011362	0.013707589	0.82958807
PC14	0.000104561	0.012614592	0.842202661
PC15	9.54E-05	1.15E-02	8.54E-01
PC16	8.65E-05	1.04E-02	8.64E-01
PC17	7.70E-05	9.29E-03	8.73E-01
PC18	7.32E-05	8.83E-03	8.82E-01
PC19	6.41E-05	7.74E-03	8.90E-01
PC20	5.78E-05	6.97E-03	8.97E-01
PC21	5.53E-05	6.67E-03	9.04E-01
PC22	4.83E-05	5.83E-03	9.09E-01
PC23	4.74886E-05	0.005729212	0.91521373
PC24	4.62E-05	5.57E-03	9.21E-01
PC25	4.26E-05	5.14E-03	9.26E-01
PC26	3.92E-05	4.73E-03	9.31E-01
PC27	3.65811E-05	0.004413288	0.935069672
PC28	3.45E-05	4.16E-03	9.39E-01

PC29	3.22112E-05	0.003886086	0.943116922
PC30	3.20E-05	3.87E-03	9.47E-01
PC31	2.98E-05	3.60E-03	9.51E-01
PC32	2.44E-05	2.94E-03	9.54E-01
PC33	2.37E-05	2.86E-03	9.56E-01
PC34	2.09E-05	2.52E-03	9.59E-01
PC35	1.99E-05	2.40E-03	9.61E-01
PC36	1.90E-05	2.29E-03	9.64E-01
PC37	1.86E-05	2.25E-03	9.66E-01
PC38	1.81E-05	2.18E-03	9.68E-01
PC39	1.70795E-05	0.002060538	0.970079507
PC40	1.45161E-05	0.001751279	0.971830786
PC41	1.35E-05	1.62E-03	9.73E-01
PC42	1.28E-05	1.55E-03	9.75E-01
PC43	1.24E-05	1.50E-03	9.76E-01
PC44	1.16E-05	1.40E-03	9.78E-01
PC45	1.12E-05	1.35E-03	9.79E-01
PC46	1.07E-05	1.29E-03	9.81E-01
PC47	1.06E-05	1.27E-03	9.82E-01
PC48	9.58E-06	1.16E-03	9.83E-01
PC49	8.71E-06	1.05E-03	9.84E-01
PC50	8.43E-06	1.02E-03	9.85E-01
PC51	8.17E-06	9.86E-04	9.86E-01
PC52	8.00E-06	9.65E-04	9.87E-01
PC53	7.11E-06	8.57E-04	9.88E-01
PC54	6.85E-06	8.26E-04	9.89E-01
PC55	6.20E-06	7.47E-04	9.89E-01
PC56	6.04E-06	7.29E-04	9.90E-01
PC57	5.69E-06	6.86E-04	9.91E-01
PC58	5.27E-06	6.36E-04	9.91E-01
PC59	4.84E-06	5.84E-04	9.92E-01
PC60	4.55E-06	5.49E-04	9.93E-01
PC61	4.38E-06	5.29E-04	9.93E-01
PC62	4.08E-06	4.92E-04	9.94E-01
PC63	3.76E-06	4.53E-04	9.94E-01

PC64	3.73E-06	4.50E-04	9.95E-01
PC65	3.50E-06	4.22E-04	9.95E-01
PC66	3.36E-06	4.06E-04	9.95E-01
PC67	3.04E-06	3.67E-04	9.96E-01
PC68	3.04E-06	3.66E-04	9.96E-01
PC69	2.73E-06	3.30E-04	9.96E-01
PC70	2.43E-06	2.93E-04	9.97E-01
PC71	2.21E-06	2.67E-04	9.97E-01
PC72	1.97E-06	2.38E-04	9.97E-01
PC73	1.94E-06	2.34E-04	9.97E-01
PC74	1.76E-06	2.12E-04	9.98E-01
PC75	1.63E-06	1.97E-04	9.98E-01
PC76	1.60E-06	1.94E-04	9.98E-01
PC77	1.49E-06	1.79E-04	9.98E-01
PC78	1.37E-06	1.65E-04	9.98E-01
PC79	1.24E-06	1.49E-04	9.99E-01
PC80	1.14E-06	1.38E-04	9.99E-01
PC81	1.10E-06	1.33E-04	9.99E-01
PC82	1.03E-06	1.24E-04	9.99E-01
PC83	9.03E-07	1.09E-04	9.99E-01
PC84	8.56E-07	1.03E-04	9.99E-01
PC85	7.67E-07	9.26E-05	9.99E-01
PC86	7.42E-07	8.95E-05	9.99E-01
PC87	6.79E-07	8.19E-05	9.99E-01
PC88	5.88E-07	7.10E-05	9.99E-01
PC89	5.76E-07	6.95E-05	1.00E+00
PC90	4.48E-07	5.40E-05	1.00E+00
PC91	4.06E-07	4.90E-05	1.00E+00
PC92	3.84E-07	4.63E-05	1.00E+00
PC93	3.36E-07	4.05E-05	1.00E+00
PC94	3.06E-07	3.70E-05	1.00E+00
PC95	2.43E-07	2.93E-05	1.00E+00
PC96	2.11E-07	2.55E-05	1.00E+00
PC97	2.04E-07	2.46E-05	1.00E+00
PC98	1.82E-07	2.20E-05	1.00E+00

PC99	1.47E-07	1.77E-05	1.00E+00
PC100	1.34E-07	1.62E-05	1.00E+00
PC101	1.23E-07	1.48E-05	1.00E+00
PC102	9.92E-08	1.20E-05	1.00E+00
PC103	9.18E-08	1.11E-05	1.00E+00
PC104	7.33E-08	8.84E-06	1.00E+00
PC105	6.42E-08	7.74E-06	1.00E+00
PC106	5.71E-08	6.88E-06	1.00E+00
PC107	3.75E-08	4.53E-06	1.00E+00
PC108	3.20E-08	3.87E-06	1.00E+00
PC109	2.34E-08	2.82E-06	1.00E+00
PC110	1.49E-08	1.80E-06	1.00E+00
PC111	1.36E-08	1.64E-06	1.00E+00
PC112	8.12E-09	9.80E-07	1.00E+00
PC113	4.54E-09	5.48E-07	1.00E+00
PC114	1.55E-09	1.87E-07	1.00E+00
PC115	4.60E-19	5.55E-17	1.00E+00
PC116	8.23E-20	9.93E-18	1.00E+00
PC117	2.52E-20	3.04E-18	1.00E+00
PC118	4.54E-21	5.48E-19	1.00E+00

Supplementary Table 2. Functional principal component analysis eigenvalues. Abbreviation: fPC, functional principal component.

**Functional Principal Component**	**eigenvalue**	**Proportion Of Variation**	**Cumulative Variation**
fPC1	2.96487647	37.0609559	37.06096
fPC2	1.88331029	23.5413786	60.60233
fPC3	0.91079789	11.3849737	71.98731
fPC4	0.8403576	10.50447	82.49178
fPC5	0.63520146	7.9400183	90.4318
fPC6	0.4291517	5.3643962	95.79619
fPC7	0.3083674	3.8545925	99.65079
fPC8	0.02793718	0.3492147	100

Supplementary Table 3. Functional principal component analysis character loadings.

Abbreviation: fPC, functional principal component. MA, mechanical advantage.

**Functional Characters**	**Functional Principal Components**							
	fPC1	fPC2	fPC3	fPC4	fPC5	fPC6	fPC7	fPC8
C1. Mean Anterior MA	0.4888	0.2748	-0.0185	0.2044	0.0482	-0.2974	-0.4591	0.5847
C2. Mean Posterior MA	0.0417	0.6794	0.1057	0.1756	-0.2916	0.0708	-0.2793	-0.5715
C3. Opening MA	-0.1404	0.4650	0.2329	-0.4273	0.7207	0.0162	0.0662	0.0563
C4. Maximum Aspect Ratio	0.4584	0.2533	-0.1062	0.2033	0.0148	-0.1814	0.7988	-0.0430
C5. Relative Toothrow Length	-0.4294	0.3777	0.0535	0.0587	-0.3847	0.3683	0.2403	0.5703
C6. Relative Symphyseal Length	0.4494	-0.0183	-0.3250	-0.1082	0.1306	0.8098	-0.0867	-0.0064
C7. Symphyseal Angle	-0.3136	0.0032	-0.2228	0.7820	0.4758	0.1084	-0.0357	-0.0319
C8. Quadrate Articular Offset	0.2117	-0.1985	0.8745	0.2770	0.0394	0.2649	0.0475	0.0159

Supplementary Table 4. PERMANOVA results for shape differences between synapsid carnivore clades. Abbreviations: BIA, Biarmosuchia. Bonf, Bonferroni-corrected. CYN, Cynodontia. DIN, Dinocephalia. EOT, Eothyrididae. GRG, Gorgonopsia, OPH, Ophiacodontidae. SPH, Sphenacodontia. SS, Sum of squares. THR, Therocephalia. VAR, Varanopidae.

Clade	Result	EOT	VAR	OPH	SPH	BIA	DIN	GRG	THR	CYN
**EOT**	**Raw P**		0.0319	0.3284	0.0244	0.0345	0.0158	0.0024	0.0067	0.0176
	**Bonf. P**		1	1	0.8784	1	0.5688	0.0864	0.2412	0.6336
	**F**		2.178	1.222	2.686	2.501	3.517	3.565	2.836	3.725
**VAR**	**Raw P**			0.0128	0.0001	0.0001	0.0001	0.0001	0.0001	0.0001
	**Bonf. P**			0.4608	0.0036	0.0036	0.0036	0.0036	0.0036	0.0036
	**F**			2.737	10.01	11.23	15.28	19.15	14.51	13.98
**OPH**	**Raw P**				0.0006	0.0012	0.0011	0.0001	0.0001	0.0007
	**Bonf. P**				0.0216	0.0432	0.0396	0.0036	0.0036	0.0252
	**F**				4.79	5.404	8.398	7.946	5.2	6.914
**SPH**	**Raw P**					0.0006	0.0001	0.0001	0.0001	0.0002
	**Bonf. P**					0.0216	0.0036	0.0036	0.0036	0.0072
	**F**					3.826	5.428	6.885	7.836	3.785
**BIA**	**Raw P**						0.1392	0.0001	0.0074	0.0004
	**Bonf. P**						1	0.0036	0.2664	0.0144
	**F**						1.5	3.999	2.558	3.469
**DIN**	**Raw P**							0.0003	0.0007	0.0001
	**Bonf. P**							0.0108	0.0252	0.0036
	**F**							3.636	3.763	3.771
**GRG**	**Raw P**								0.0001	0.0002
	**Bonf. P**								0.0036	0.0072
	**F**								6.15	3.682
**THE**	**Raw P**									0.0007
	**Bonf. P**									0.0252
	**F**									3.483
**CYN**	**Raw P**									
	**Bonf. P**									
	**F**									
**Total**	**Total SS**	1.0								
	**Within-group SS**	0.7								
	**P**	0.0001								
	**F**	6.107								

Supplementary Table 5. PERMANOVA results for functional differences between synapsid carnivore clades. Abbreviations: BIA, Biarmosuchia. Bonf, Bonferroni-corrected. CYN, Cynodontia. DIN, Dinocephalia. EOT, Eothyrididae. GRG, Gorgonopsia, OPH, Ophiacodontidae. SPH, Sphenacodontia. SS, Sum of squares. THR, Therocephalia. VAR, Varanopidae.

Clade	Result	EOT	VAR	OPH	SPH	BIA	DIN	GRG	THR	CYN
EOT	Raw P		0.3426	0.3843	0.4721	0.0243	0.0442	0.002	0.3426	0.0947
	Bonf. P		1	1	1	0.8748	1	0.072	0.0792	1
	F		1.133	0.9989	0.8613	3.132	2.367	6.851	4.074	2.598
VAR	Raw P			0.2115	0.0003	0.0001	0.0001	0.0001	0.0001	0.0001
	Bonf. P			1	0.0108	0.0036	0.0036	0.0036	0.0036	0.0036
	F			1.476	7.288	10.76	9.808	33.82	17.55	13.6
OPH	Raw P				0.0115	0.0002	0.0002	0.0001	0.0001	0.0021
	Bonf. P				0.414	0.0072	0.0072	0.0036	0.0036	0.0756
	F				3.208	7.373	6.274	20.08	10.29	8.906
SPH	Raw P					0.0001	0.0001	0.0001	0.0001	0.0032
	Bonf. P					0.0036	0.0036	0.0036	0.0036	0.1152
	F					9.477	7.64	22.85	15.84	4.409
BIA	Raw P						0.7373	0.0001	0.0105	0.0002
	Bonf. P						1	0.0036	0.378	0.0072
	F						0.5847	6.734	3.341	7.908
DIN	Raw P							0.0005	0.0228	0.0012
	Bonf. P							0.018	0.8208	0.0432
	F							4.685	2.725	5.924
GRG	Raw P								0.0001	0.0001
	Bonf. P								0.0036	0.0036
	F								9.974	9.065
THE	Raw P									0.0001
	Bonf. P									0.0036
	F									8.26
CYN	Raw P									
	Bonf. P									
	F									
Total	Total SS	976								
	Within-group SS	590								
	P	0.0001								
	F	9.239								

Supplementary Table 6. Internal validation statistics for cluster configurations. Results for analyses run with all taxa, and each identified feeding functional group. Abbreviations: FFG, Feeding functional group. MDB, mean distance between cluster centroids. MDC, mean distance from cluster centroid. PAM, partition around medoids. pF Value, pseudo F value. WSS, within cluster sum of squares.

**Internal Validation Index**	**Data**	**Clustering Methods**		
		**Hierarchical**	**K-means**	**PAM**
**WSS**	All Taxa	914.7134	596.4908	597.3226
	FFG1	145.131	145.131	145.131
	FFG2	186.527	184.6536	201.9363
	FFG3	113.1085	111.3767	133.4041
**MDC**	All Taxa	3.727941	3.023226	3.024016
	FFG1	2.574005	2.574005	2.574005
	FFG2	2.76468	2.749539	2.87168
	FFG3	2.589147	2.578228	2.800941
**MDB**	All Taxa	3.854758	4.199812	4.202307
	FFG1	3.160952	3.160952	3.160952
	FFG2	3.61181	3.622567	3.524632
	FFG3	3.22876	3.254656	3.048589
**pF Value**	All Taxa	1.034018	1.389182	1.389645
	FFG1	1.228029	1.228029	1.228029
	FFG2	1.306412	1.317518	1.227377
	FFG3	1.247036	1.262362	1.088416
**Dunn Index**	All Taxa	0.1010731	0.1636738	0.1883225
	FFG1	0.2438311	0.2438311	0.2438311
	FFG2	0.2481868	0.2283256	0.264629
	FFG3	0.3003869	0.2486065	0.3155862

Supplementary Table 7. External validation statistics for different cluster configurations. Examination of the agreement between cluster groups and phylogenetic groups, at broad and higher taxonomic resolution (**See Supplementary Data SX**), during identification of feeding functional group and subgroups. Abbreviations: FFG, Feeding functional group. PAM, partition around medoids.

External Index	Data	Hierarchical		K-means		PAM	
		**Broad**	**Higher**	**Broad**	**Higher**	**Broad**	**Higher**
**Corrected Rand Index**	All Taxa	0.1852451	0.1163629	0.1852451	0.02217388	0.04268881	0.02706362
	FFG1	-0.03047528	0.01332104	-0.03047528	0.01332104	-0.03047528	0.01332104
	FFG2	0.2353576	0.142697	0.2353576	0.142697	0.1624334	0.1006176
	FFG3	0.08697908	0.09867758	0.04132803	0.053041	0.003285492	0.00740442
**Meila's VI Index**	All Taxa	2.1159	2.557417	2.813495	3.269911	2.751141	3.21913
	FFG1	1.860208	2.256557	1.860208	2.256557	1.860208	2.256557
	FFG2	1.45274	1.924394	1.45274	1.924394	1.663936	2.057649
	FFG3	1.969793	1.891421	1.992029	1.913657	1.999068	1.929383

Supplementary Table 8. PERMANOVA results for significant differences between synapsid carnivore mandibular shape and function between feeding functional groups. Abbreviations: Bonf. Bonferroni corrected. SS, Sum of squares.

**Feeding Functional Group**	**Result**	**Power Specialists**		**Speed Specialists**	
		**Shape**	**Function**	**Shape**	**Function**
**Raptorial Specialists**	**Raw P**	0.0001	0.0001	0.0001	0.0001
	**Bonf. P**	0.0003	0.0003	0.0003	0.0003
	**F**	25.87	47.66	15.76	37.14
**Power Specialists**	**Raw P**			0.0001	0.0001
	**Bonf. P**			0.0003	0.0003
	**F**			8.171	26.71
**Total**		**Shape**		**Function**	
	**Total SS**	1.003		976	
	**Within-group SS**	0.7867		602.3	
	**P**	0.0001		0.0001	
	**F**	16.35		36.92	

Supplementary Table 9. PERMANOVA results for significant differences in synapsid carnivore mandibular shape between feeding functional subgroups. Abbreviations: Bonf. Bonferroni corrected. SS, Sum of squares.

Feeding Functional Group	Result	Gracile Gripper	Forceful Gripper	Power Bite Specialist	Shearing Bite Specialist	Deep Shearing Specialist	Grip and Rip Attacker	Rapid Light Attacker
Gracile Gripper	Raw P		0.0001	0.0001	0.0001	0.0001	0.0001	0.0001
	Bonf. P		0.0021	0.0021	0.0021	0.0021	0.0021	0.0021
	F		5.884	18.07	23.04	20.58	19.96	7.076
Forceful Gripper	Raw P			0.0001	0.0001	0.0001	0.0001	0.0001
	Bonf. P			0.0021	0.0021	0.0021	0.0021	0.0021
	F			6.679	10.51	10.63	8.666	5.742
Power Bite Specialist	Raw P				0.0021	0.0001	0.0001	0.0001
	Bonf. P				0.0441	0.0021	0.0021	0.0021
	F				2.592	3.553	5.526	9.809
Shearing Bite Specialist	Raw P					0.0001	0.0006	0.0001
	Bonf. P					0.0021	0.0126	0.0021
	F					3.273	2.761	7.566
Deep Shearing Specialist	Raw P						0.0001	0.0001
	Bonf. P						0.0021	0.0021
	F						6.195	9.327
Grip and Rip Attacker	Raw P							0.0001
	Bonf. P							0.0021
	F							4.612
Rapid Light Attacker	Raw P							
	Bonf. P							
	F							
Total				Shape				
	Total SS			1.003				
	Within-group SS			0.6882				
	P			0.0001				
	F			8.764				

Supplementary Table 10. PERMANOVA results for significant differences in synapsid carnivore mandibular function between feeding functional subgroups. Abbreviations: Bonf. Bonferroni corrected. SS, Sum of squares.

Feeding Functional Group	Result	Gracile Gripper	Forceful Gripper	Power Bite Specialist	Shearing Bite Specialist	Deep Shearing Specialist	Grip and Rip Attacker	Rapid Light Attacker
Gracile Gripper	Raw P		0.0001	0.0001	0.0001	0.0001	0.0001	0.0001
	Bonf. P		0.0021	0.0021	0.0021	0.0021	0.0021	0.0021
	F		10.28	28.13	49.37	52.98	33.52	17.4
Forceful Gripper	Raw P			0.0001	0.0001	0.0001	0.0001	0.0001
	Bonf. P			0.0021	0.0021	0.0021	0.0021	0.0021
	F			10.67	24.84	32.11	28.47	24.84
Power Bite Specialist	Raw P				0.0001	0.0001	0.0001	0.0002
	Bonf. P				0.0021	0.0021	0.0021	0.0042
	F				11.47	10.21	23.05	29.93
Shearing Bite Specialist	Raw P					0.0001	0.0001	0.0001
	Bonf. P					0.0021	0.0021	0.0021
	F					23.89	13.04	23.89
Deep Shearing Specialist	Raw P						0.0001	0.0001
	Bonf. P						0.0021	0.0021
	F						15.05	31.97
Grip and Rip Attacker	Raw P							0.0001
	Bonf. P							0.0021
	F							8.971
Rapid Light Attacker	Raw P							
	Bonf. P							
	F							
Total				Function				
	Total SS			976				
	Within-group SS			445.4				
	P			0.0001				
	F			22.83				

Supplementary Table 11. Support for different macroevolutionary models of mandibular functional disparity evolution. Weighted Akaike Information Criterion and log-likelihood values for each model. Abbreviations: BIA, Biarmosuchia. BM, Brownian motion. BSL SYN, Basal-most synapsids. CYN, Cynodontia. DIN, Dinocephalia. EB, Early Burst. GRG, Gorgonopsia, Log. Lik, Log likelihood. OU, Ornstein-Uhlenbeck. SPH, (Non-therapsid) Sphenacodontia. THR, Therocephalia. W. AIC, Weighted Akaike Information Criterion.

Models			BM	EB	OU	Stasis	Trend
**Taxonomic Groups**	**BSL SYN**	**W. AIC**	0.36	0	0.08	0	0.57
		**Log. Lik.**	-18.8	-34.7	-18.8	-33.7	-16.8
	**SPH**	**W. AIC**	0.4	0.01	0.05	0.001	0.55
		**Log. Lik.**	-10.4	-11.8	-10.4	-16.5	-7.9
	**BIA**	**W. AIC**	0	81.06	3.86	0.77	3.07
		**Log. Lik.**	0.49	0	0.07	0.33	0.11
	**DIN**	**W. AIC**	0.4	0	0.01	0.02	0.57
		**Log. Lik.**	-14	-114	-14	-17	-11
	**GRG**	**W. AIC**	11.25	41.11	15.85	14.64	0
		**Log. Lik.**	0.004	0	0	0.001	0.1
	**THE**	**W. AIC**	0.03	0	0.01	0	0.97
		**Log. Lik.**	-14.5	-31.7	-14.1	-19	-8.8
	**CYN**	**W. AIC**	6.56	19.85	10	0	16.54
		**Log. Lik.**	0.04	0	0.01	0.96	0

Supplementary Table 12. PERMANOVA results for significant differences between synapsid carnivore mandibular shape and function between geological stages. Abbreviations: ART, Artinskian. ASL, Asselian. Bonf. Bonferroni corrected. CAP, Capitanian. CHX, Changhsingian. GZH, Gzhelian. IND, Induan. KAS, Kasimovian. KUN, Kungurian. SAK, Sakmarian. ROA, Roadian. WOR, Wordian. WUC, Wuchiapingian.

	**Shape**			**Function**		
**Stage Transitions**	P	Bonf. P	F	P	Bonf. P	F
KAS>GZH	0.3003	1	1.434	0.205	1	1.766
GZH>ASL	0.285	1	1.187	0.2056	1	1.527
ASL>SAK	0.7297	1	0.555	0.6513	1	0.6113
SAK>ART	0.9754	1	0.2388	0.9582	1	0.1822
ART>KUN	0.9812	1	0.2344	0.8697	1	0.3373
KUN>ROA	0.9027	1	0.3793	0.6179	1	0.6559
ROA>WOR	0.1649	1	1.512	0.1686	1	1.544
WOR>CAP	0.3566	1	1.045	0.3913	1	1.019
CAP>WUC	0.0723	1	1.641	0.0153	1	2.905
WUC>CHX	0.9998	1	0.2543	0.8109	1	0.4438
CHX>IND	0.1737	1	1.334	0.1181	1	1.812
Total	0.0001	-	3.071	0.0001	-	5.384
Total sum of squares:	1.491			1452		
Within-group sum of squares:	1.234			1065		

Supplementary Table 13. Mann-Whitney U test results for significant body size changes in synapsid carnivores between geological stages. Abbreviations: ART, Artinskian. ASL, Asselian. Bonf. Bonferroni corrected. CAP, Capitanian. CHX, Changhsingian. GZH, Gzhelian. IND, Induan. KAS, Kasimovian. KUN, Kungurian. SAK, Sakmarian. ROA, Roadian. WOR, Wordian. WUC, Wuchiapingian.

	Body Size		
**Stage>Transitions**	P	Bonf. P	U Value
KAS>GZH	0.7728	1	3
GZH>ASL	0.2997	1	4.5
ASL>SAK	0.9362	1	17
SAK>ART	0.786	1	27
ART>KUN	0.5329	1	32.5
KUN>ROA	0.9187	1	11
ROA>WOR	0.2565	1	11.5
WOR>CAP	0.0322	1	87.5
CAP>WUC	0.5651	1	431
WUC>CHX	0.4066	1	491.5
CHX>IND	0.0006	0.03847	46

Supplementary Table 14. Support for different models of ancestral trait estimation of feeding functional subgroup. Akaike Information Criterion and log-likelihood values for each model. Abbreviations: AICc, Second Order Akaike Information Criterion. Log. Lik, Log likelihood. W. AICc, Weighted Second Order Akaike Information Criterion.

**Trait Transition Model**	**Log. Lik**	**AICc**	**W. AICc**
**Equal Rates**	-186.39	374.81	0.27
**Symmetrical Rates**	-149.20	373.07	0.64
**All Rates Different**	-136.72	493.64	0.00
**Asymmetrical Rates**	-186.36	376.83	0.10

Supplementary Figures.

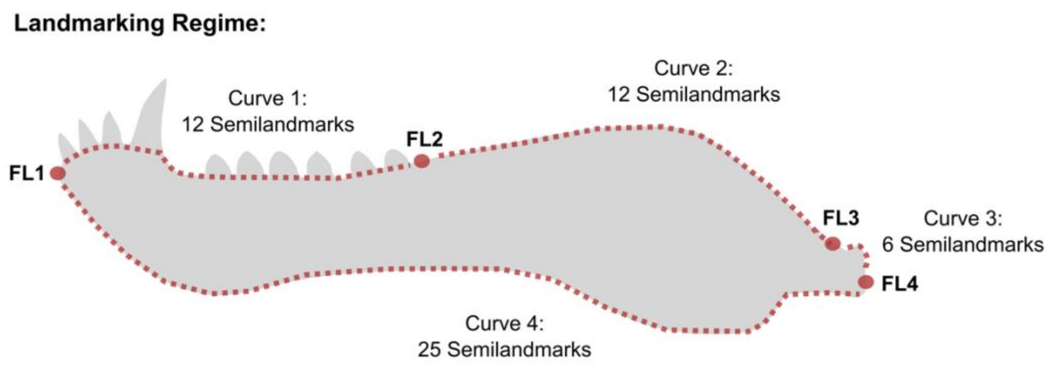

Supplementary Figure 1. Landmarking regime for shape geometric morphometric analyses. morphospaces. Fixed landmarks (four) and semi-landmark curves (totalling 55 semi-landmarks) represented by circles and dotted lines, respectively. Abbreviations: FL, Fixed landmark.

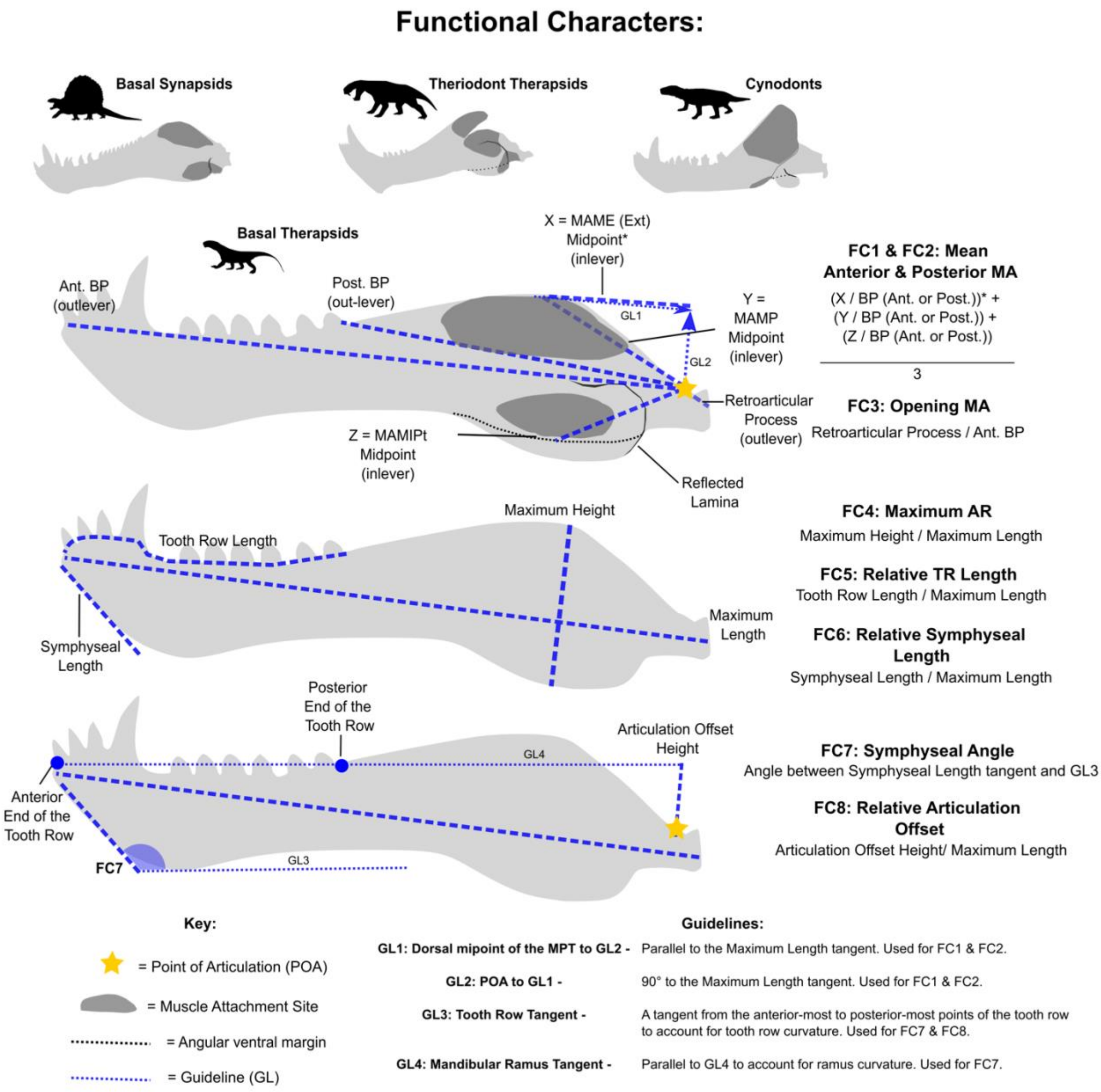

Supplementary Figure 2. Functional linear measurement and character guide. Guide illustrating the linear measurements recorded from the lateral view images of synapsid mandibles, and how these measurements were used to calculate the functional character data. Based on functional characters used by Singh et al.[1], Functional Characters = FC: 1). Mean Anterior Mechanical Advantage, 2). Mean Posterior Mechanical Advantage, 3). Opening Mechanical Advantage, 4). Relative Maximum Aspect Ratio, 5). Relative Toothrow Length, 6). Relative Symphysis Length, 7). Symphyseal angle, 8). Relative Articulation Offset. Abbreviations: Ant, Anterior. BP, Biting point. Ext, External. MAME, Adductor mandibulae externus muscle group. MAMP, Adductor mandibulae posterior muscle group. MAMIPt, Adductor mandibulae internus pterygoideus muscle group. Post, Posterior. Biarmosuchia/basal therapsid silhouette by Dmitry Bogdanov (vectorized by T. Michael Keesey); All

other silhouettes created by S.A.S., but some are vectorised from artwork by Felipe Alves Elias (https://www.paleozoobr.com/), available for academic use with attribution.

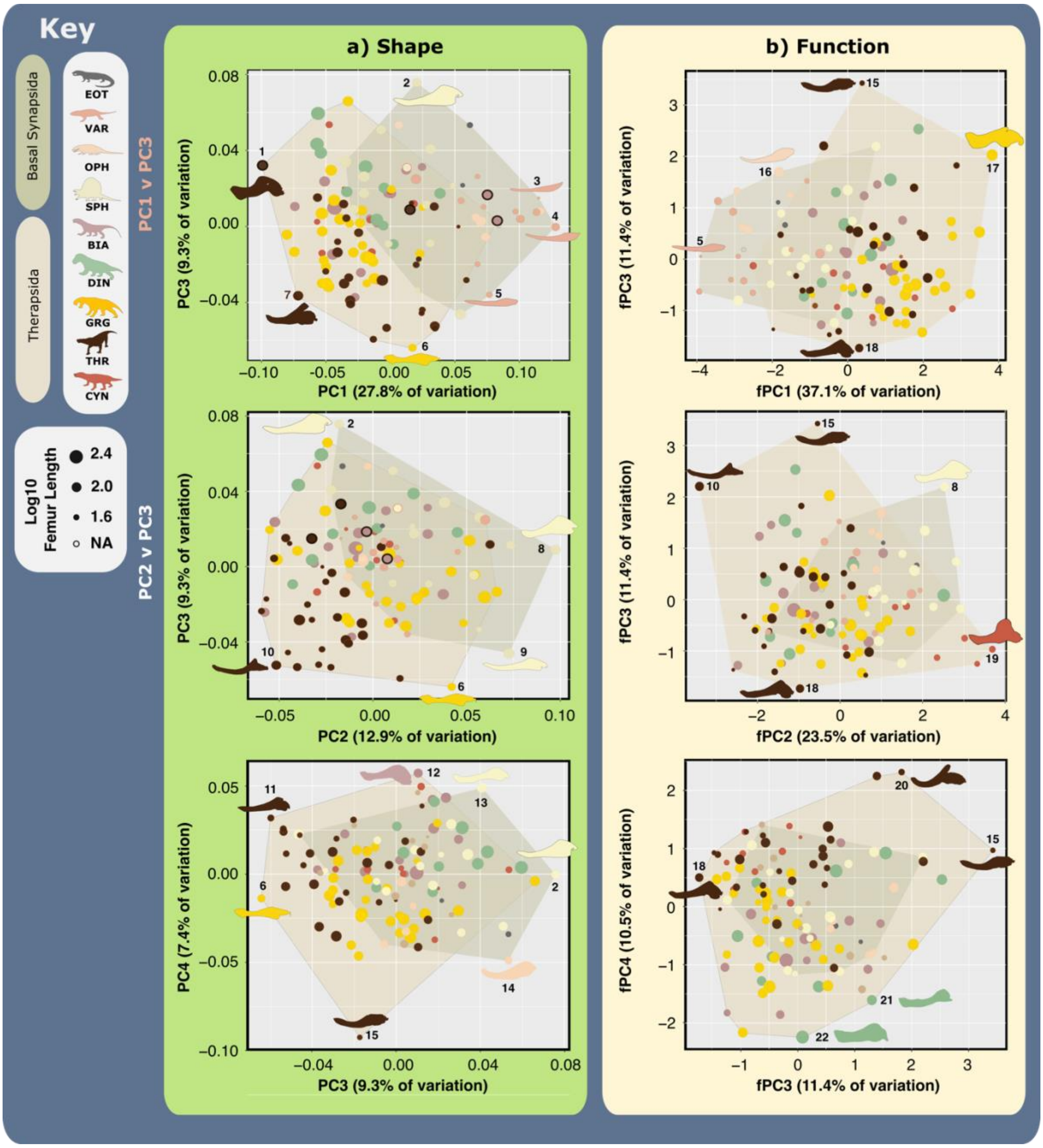

Supplementary Figure 3. Additional carnivorous synapsid mandibular morphofunctional morphospaces. Additional morphospaces showing distribution of taxa across principal component three. a) Mandibular shape morphospaces. b) Mandibular functional morphospaces. Overall basal synapsid and therapsid morphospace occupation shown through shaded convex hulls. Point size represents Log_{10}(mm) femur length. N=122. Mandible silhouettes: 1. *Purlovia maxima*, 2. *Dimetrodon natalis*, 3. *Archaeovenator hamiltonensis*, 4. *Varanodon agilis*, 5. *Mycterosaurus*

longiceps, 6. *Aleurosaurus felinus*, 7. *Glanosuchus macrops*, 8. *Sphenacodon ferox*, 9. *Secodontosaurus obtusidens*, 10. *Lycideops longiceps*, 11. *Olivierosuchus parringtoni*, 12. *Leucocephalus wewersi*, 13. *Dimetrodon loomisi*, 14. *Stereorachis dominans*, 15. *Ophidostoma tartarinovi*, 16. *Ophiacodon retroversus*, 17. *Dinogorgon rubidgei*, 18. *Viatokosuchus sumini*, 19. *Vetusodon elikhulu*. 20. *Mirotenthes digitipes*, 21. *Australosyodon nyaphuli*, 22. *Anteosaurus magnificus*. Abbreviations: BIA, Biarmosuchia. CYN, Cynodontia. DIN, Dinocephalia. EOT, Eothyrididae. fPC, functional principal component. GRG, Gorgonopsia, OPH, Ophiacodontidae. PC, Principal component. SPH, Sphenacodontia (non-therapsid). THR, Therocephalia. VAR, Varanopidae. Biarmosuchia, Dinocephalia and Therocephalia silhouettes by Dmitry Bogdanov (vectorized by T. Michael Keesey); All other silhouettes created by S.A.S., but some are vectorised from artwork by Felipe Alves Elias (https://www.paleozoobr.com/), available for academic use with attribution.

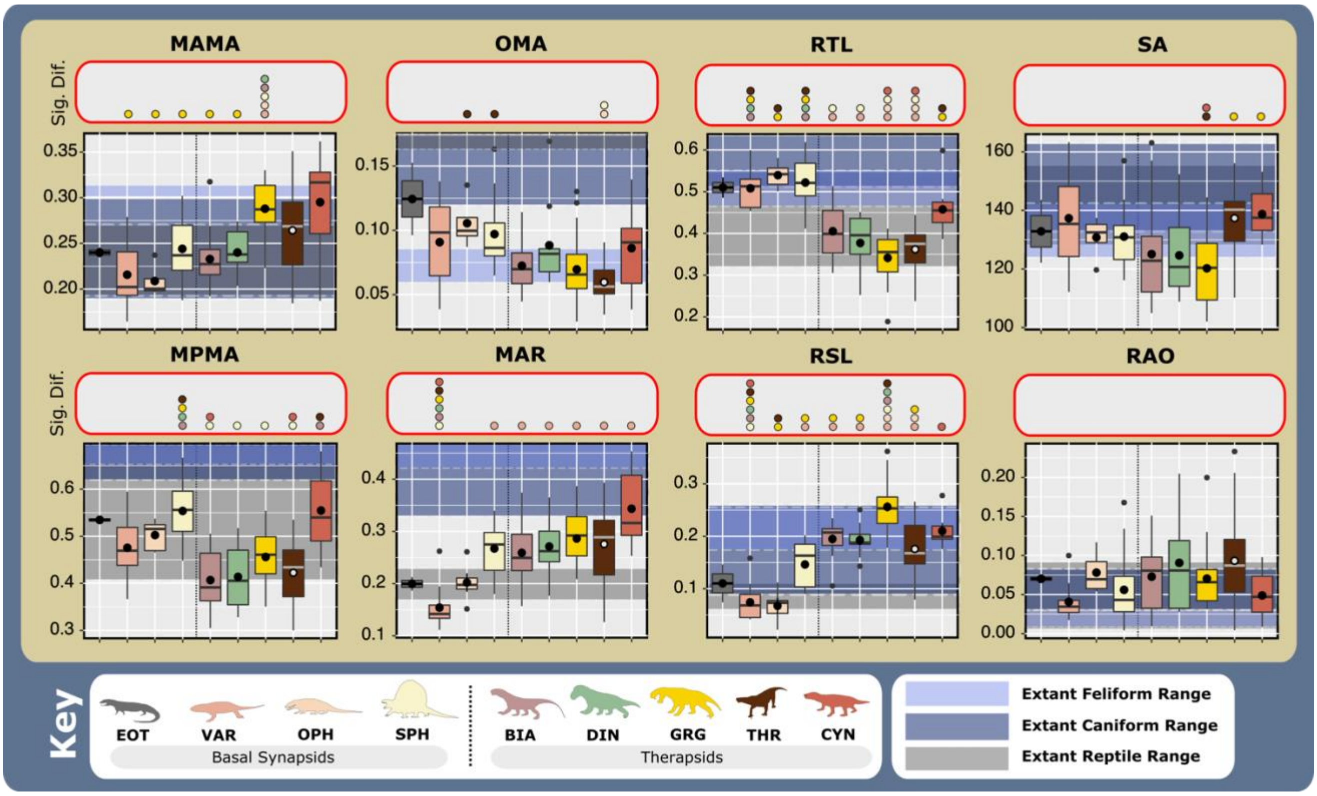

Supplementary Figure 4. Carnivorous synapsid mandibular functional character distributions. Boxplots illustrating group ranges across all functional characters with extant mammalian and reptile ranges (shaded). Statistically significant differences (<0.05) between groups highlighted by clade colour-coded circles. N = 122. Abbreviations: BIA, Biarmosuchia. CYN, Cynodontia. DIN, Dinocephalia. EOT, Eothyrididae. GRG, Gorgonopsia, MAMA, Mean anterior mechanical advantage. MAR, Maximum aspect ratio. MPMA, Mean posterior mechanical advantage. OMA, Opening

mechanical advantage. OPH, Ophiacodontidae. RAO, Relative articulation offset. RSL, Relative symphyseal length. RTL, Relative toothrow length. SA, Symphyseal angle. SPH, Sphenacodontia (non-therapsid). THR, Therocephalia. VAR, Varanopidae. Biarmosuchia, Dinocephalia and Therocephalia silhouettes by Dmitry Bogdanov (vectorized by T. Michael Keesey); All other silhouettes created by S.A.S., but some are vectorised from artwork by Felipe Alves Elias (https://www.paleozoobr.com/), available for academic use with attribution.

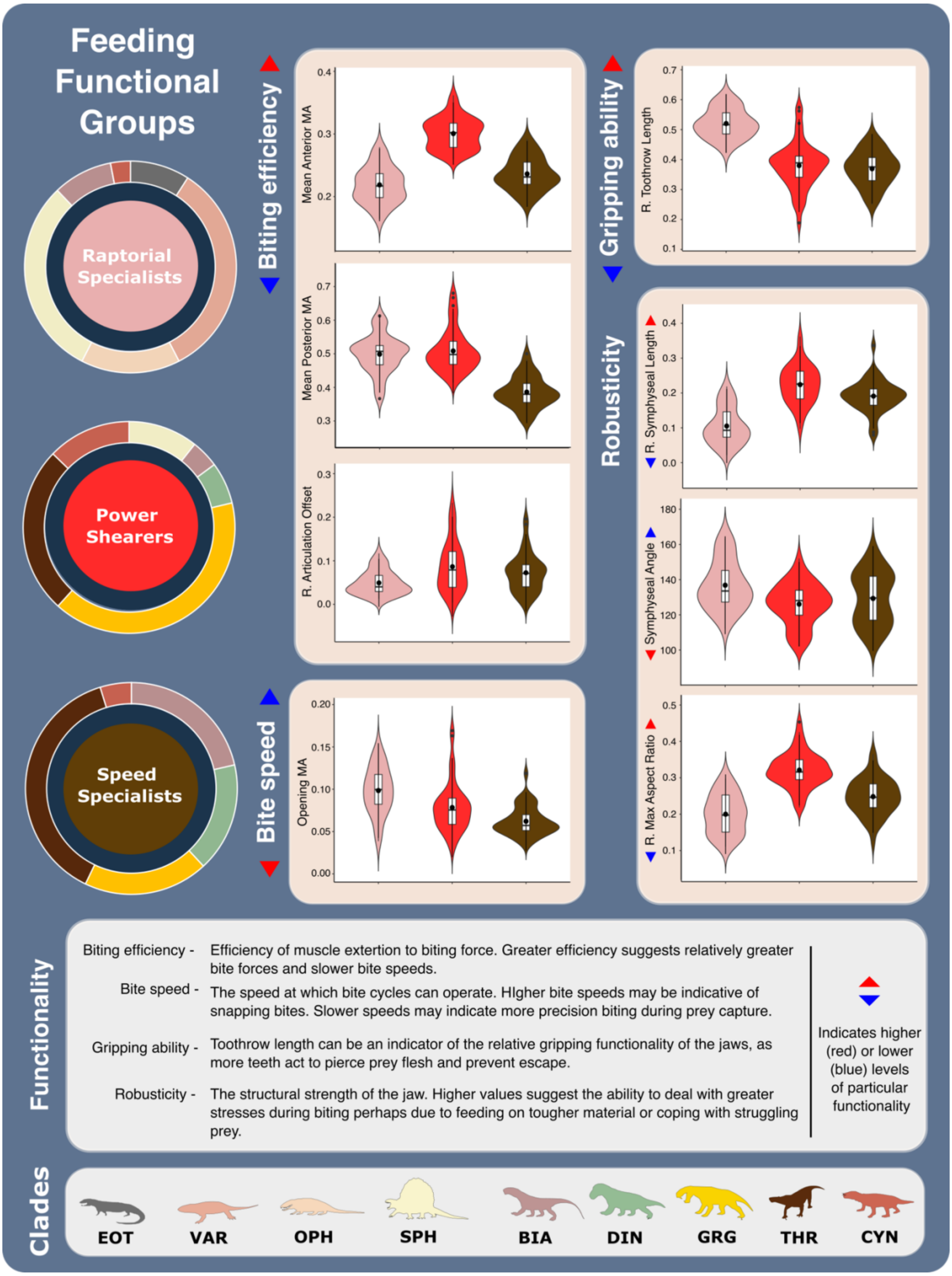

Supplementary Figure 5. Carnivorous synapsid feeding functional group mandibular characteristics. The feeding functional group mandibular functional character distributions illustrated using violin and box plots. Feeding functional group compositions illustrated using ring plots

detailing relative proportions of different taxonomic groups. Mean values indicate by black dots. Coloured arrows indicate whether values increase (red) or decrease (blue) relevant mandible functionality. N=122. Abbreviations: BIA, Biarmosuchia. CYN, Cynodontia. DIN, Dinocephalia. EOT, Eothyrididae and assorted Casesauria. GRG, Gorgonopsia, MAMA, Mean anterior mechanical advantage. MAR, Maximum aspect ratio. MPMA, Mean posterior mechanical advantage. OMA, Opening mechanical advantage. OPH, Ophiacodontidae. RAO, Relative articulation offset. RSL, Relative symphyseal length. RTL, Relative toothrow length. SA, Symphyseal angle. SPH, Sphenacodontia (non-therapsid). THR, Therocephalia. VAR, Varanopidae. Biarmosuchia, Dinocephalia and Therocephalia silhouettes by Dmitry Bogdanov (vectorized by T. Michael Keesey); All other silhouettes created by S.A.S., but some are vectorised from artwork by Felipe Alves Elias (https://www.paleozoobr.com/), available for academic use with attribution.

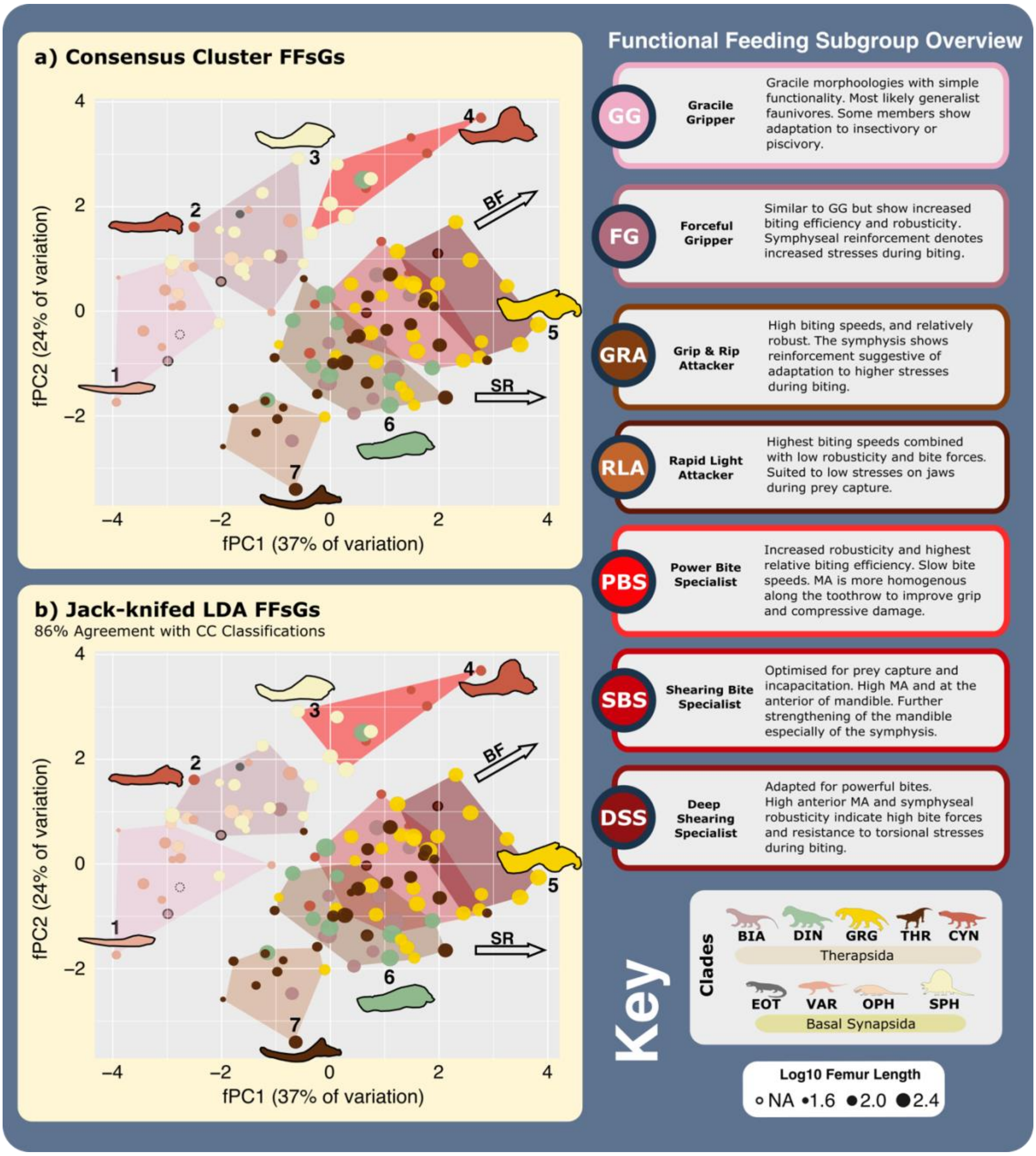

Supplementary Figure 6. Carnivorous synapsid feeding functional group validation.

a) Consensus cluster analysis feeding functional subgroups of synapsid carnivores mapped onto their functional morphospace. b) The feeding functional subgroups of synapsid carnivores resulting from a linear discriminant analysis after a jack-knifing test, mapped onto the functional morphospace**.** N=122. Jaw silhouettes: 1. *Mycterosaurus longiceps*, 2. *Procynosuchus delaharpeae*, 3. *Dimetrodon milleri*, 4. *Vetusodon elikhulu*, 5. *Dinogorgon rubidgei*, 6. *Deuterosaurus biarmicus*. 7. *Lycideops longiceps*. Abbreviations: BF, Biting force. BIA, Biarmosuchia. CC, Consensus cluster. CYN, Cynodontia. DIN, Dinocephalia. EOT, Eothyrididae and assorted Casesauria. FFsGs, Feeding functional subgroups. fPC, Functional principal components. GRG, Gorgonopsia, LDA, Linear

discriminant analysis. MA, Mechanical advantage. OPH, Ophiacodontidae. SPH, Sphenacodontia (non-therapsid). SR, Symphyseal robusticity. THR, Therocephalia. VAR, Varanopidae. Biarmosuchia, Dinocephalia and Therocephalia silhouettes by Dmitry Bogdanov (vectorized by T. Michael Keesey); All other silhouettes created by S.A.S., but some are vectorised from artwork by Felipe Alves Elias (https://www.paleozoobr.com/), available for academic use with attribution.

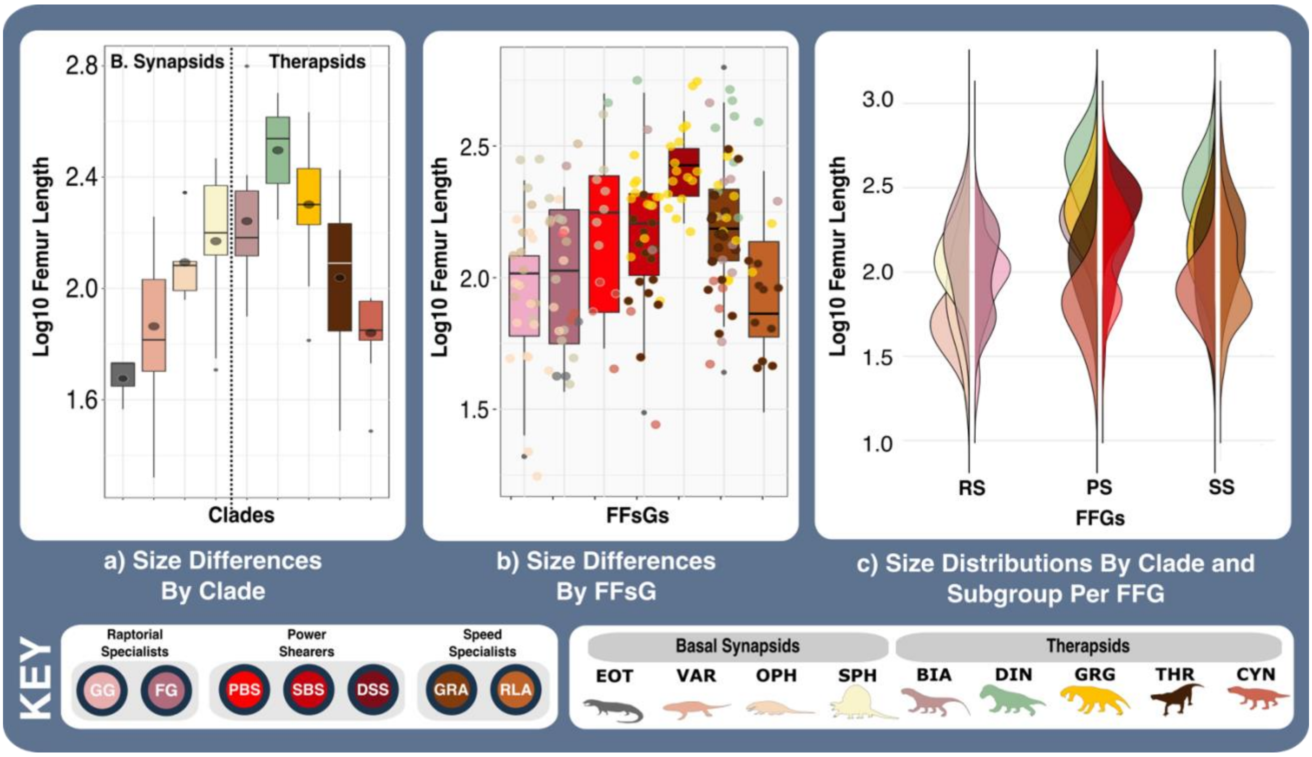

Supplementary Figure 7. Carnivorous synapsid size distributions across taxonomic and feeding functional (sub)groups. a) Boxplots illustrating body size (log_{10} femur length) distributions by taxonomic group. Dotted line represents divide between basal synapsids and therapsids. b) Boxplots illustrating body size (log_{10} femur length) distributions by feeding functional subgroup with taxonomic groups indicated by points. c) Distribution plots illustrating the body size ranges of and taxonomic groups and feeding functional subgroups within each feeding functional group. N=122. Abbreviations: BIA, Biarmosuchia. CYN, Cynodontia. DIN, Dinocephalia. DSS, Deep shearing specialist. EOT, Eothyrididae. FFGs, Feeding functional groups. FFsG, Feeding functional subgroups. FG, Forceful gripper. GG, Gracile gripper. GRA, Grip and rip attacker. GRG, Gorgonopsia, OPH, Ophiacodontidae. PBS, Power bite specialist. PS, Power shearer. RLA, Rapid light attacker. RS, Raptorial specialist. SBS, Shearing bite specialist. SPH, Sphenacodontia (non-therapsid). SS, Speed specialist. THR, Therocephalia. VAR, Varanopidae. Biarmosuchia, Dinocephalia and Therocephalia silhouettes by Dmitry Bogdanov (vectorized by T. Michael

Keesey); All other silhouettes created by S.A.S., but some are vectorised from artwork by Felipe Alves Elias (https://www.paleozoobr.com/), available for academic use with attribution.

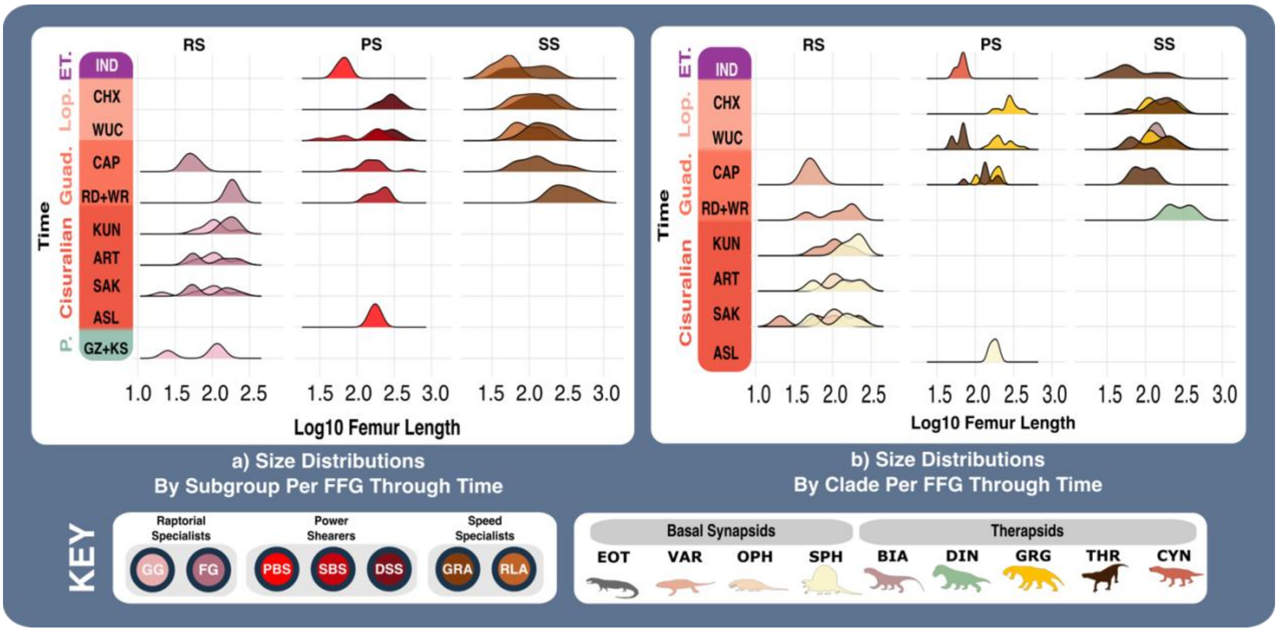

Supplementary Figure 8. Carnivorous synapsid size distributions by taxonomic and feeding functional group through the late Palaeozoic. a) Distribution plots illustrating the body size ranges of feeding functional subgroups within each feeding functional group per timebin through the late Palaeozoic. b) Distribution plots illustrating the body size ranges of different taxonomic groups within each feeding functional group per timebin through the late Palaeozoic. Body size represented by $\log_{10}$ femur length. N=122. Abbreviations: ART, Artinskian. ASL, Asselian. BIA, Biarmosuchia. CAP, Capitanian. CHX, Changhsingian. CYN, Cynodontia. DIN, Dinocephalia. DSS, Deep shearing specialist. ET, Early Triassic. EOT, Eothyrididae and assorted Casesauria. FFG, Feeding functional group. FFsG, Feeding functional subgroup. FG, Forceful gripper. GG, Gracile gripper. GRA, Grip and rip attacker. GRG, Gorgonopsia, GUAD, Guadalupian. GZH, Gzhelian. IND, Induan. LOP, Lopingian. OPH, Ophiacodontidae. PBS, Power bite specialist. PH, Primitive hypercarnivore. PS, Power shearer. RLA, Rapid light attacker. RD, Roadian. RS, Raptorial specialist. SAK, Sakmarian. SBS, Shearing bite specialist. SPH, Sphenacodontia (non-therapsid). SS, Speed specialist. THR, Therocephalia. VAR, Varanopidae. WR, Wordian. WUC, Wuchiapingian. Biarmosuchia, Dinocephalia and Therocephalia silhouettes by Dmitry Bogdanov (vectorized by T. Michael Keesey); All other silhouettes created by S.A.S., but some are vectorised from artwork by Felipe Alves Elias (https://www.paleozoobr.com/), available for academic use with attribution.

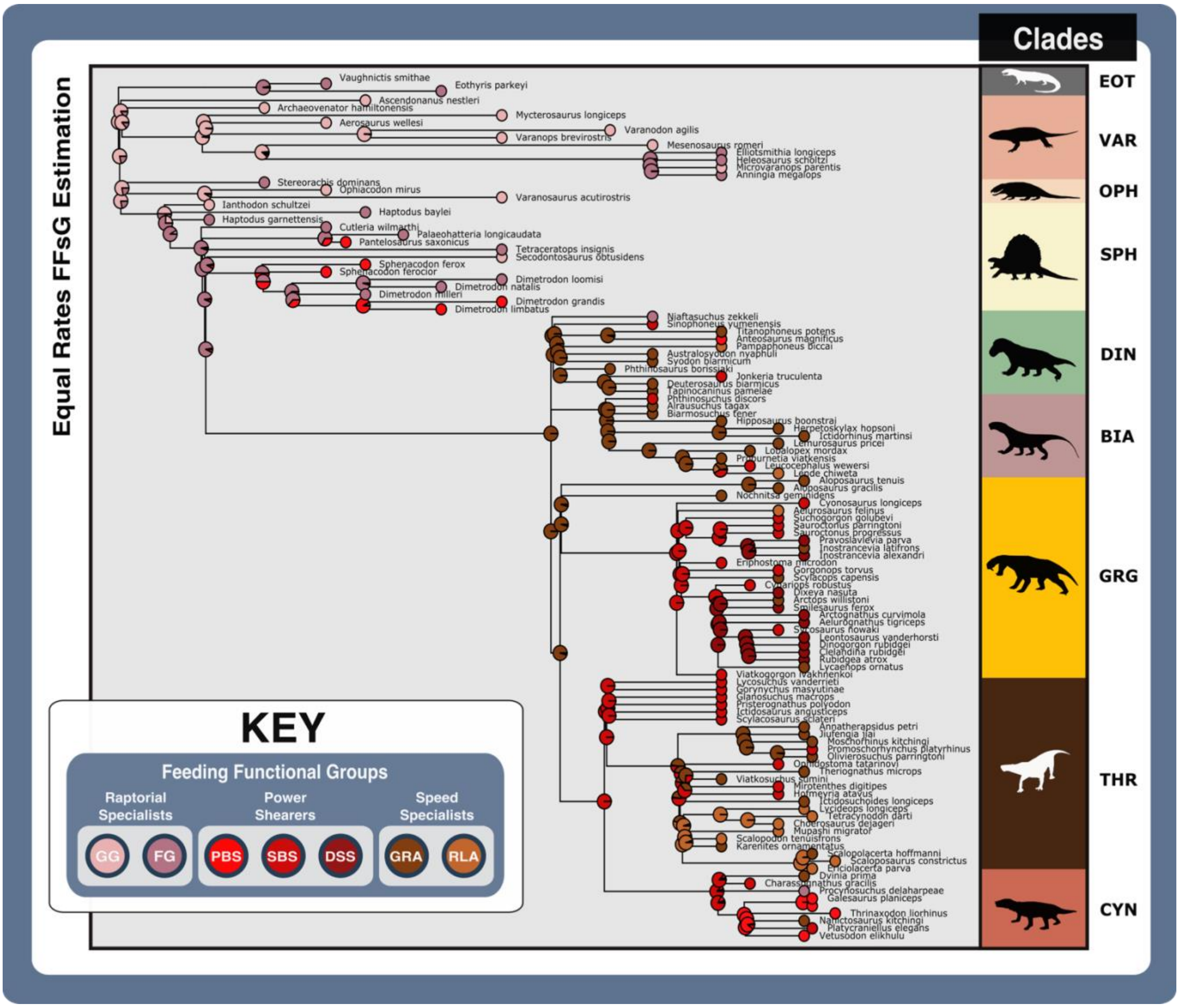

Supplementary Figure 9. Equal rates ancestral trait reconstruction of carnivorous synapsid FFsG through the late Palaeozoic. Feeding functional subgroup states cross the carnivorous synapsid phylogeny with reconstructed ancestral character state likelihoods under an equal rates model of character transitions denoted by pie charts at node positions. N=122. Abbreviations: BIA, Biarmosuchia. CYN, Cynodontia. DIN, Dinocephalia. DSS, Deep shearing specialists. EOT, Eothyrididae. FFsG, Feeding functional subgroup. FG, Forceful grippers. GG, Gracile grippers. GRA, Grip and rip attackers. GRG, Gorgonopsia, OPH, Ophiacodontidae. PBS, Power bite specialists. SBS, Shearing bite specialists. SPH, Sphenacodontia (non-therapsid). RLA, Rapid light attacker. THR, Therocephalia. VAR, Varanopidae. Biarmosuchia, Dinocephalia and Therocephalia silhouettes by Dmitry Bogdanov (vectorized by T. Michael Keesey); All other silhouettes created by S.A.S., but some are vectorised from artwork by Felipe Alves Elias (https://www.paleozoobr.com/), available for academic use with attribution.

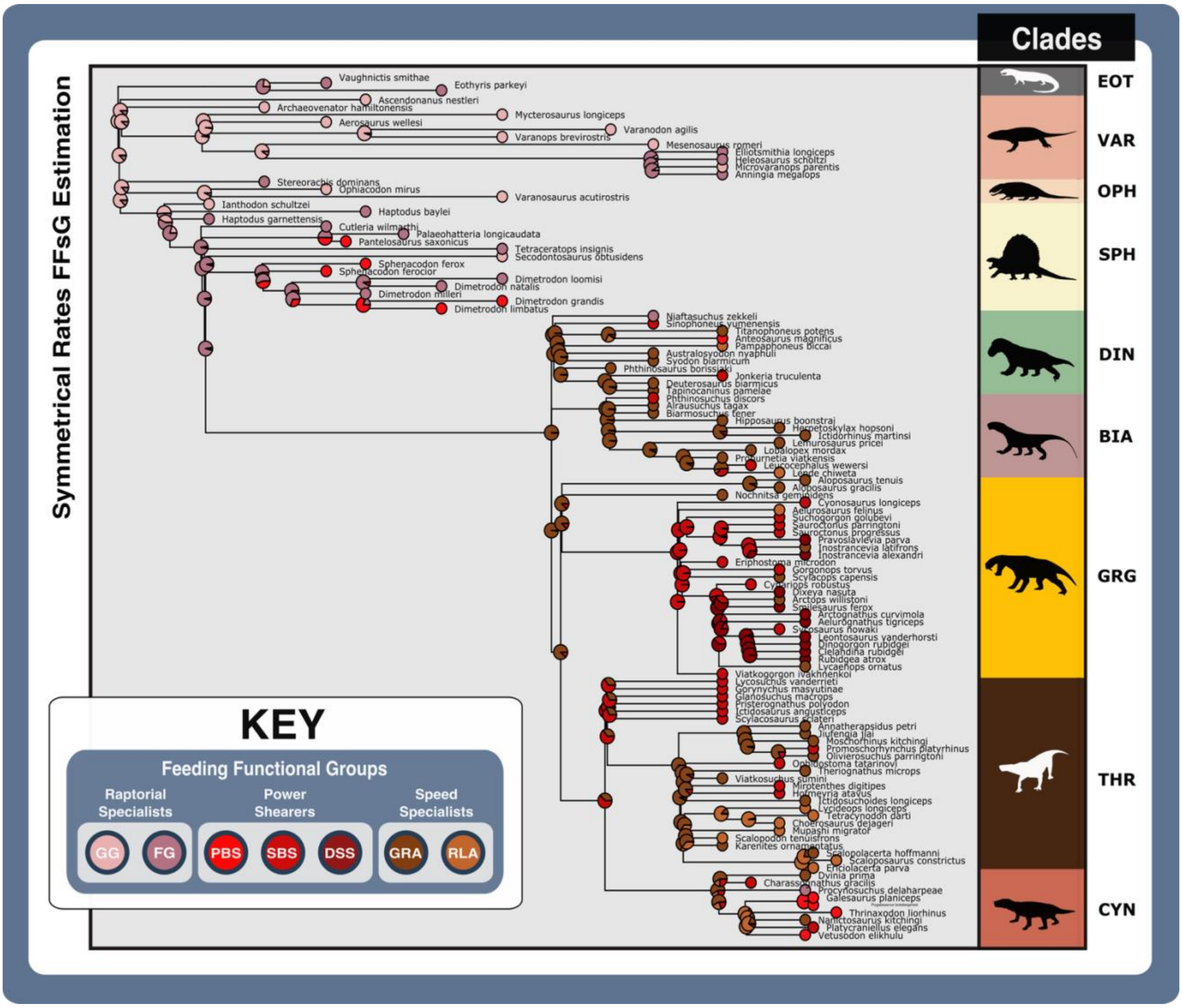

Supplementary Figure 10. Symmetrical rates ancestral trait reconstruction of carnivorous synapsid FFsG through the late Palaeozoic. Feeding functional subgroup states cross the carnivorous synapsid phylogeny with reconstructed ancestral character state likelihoods under a symmetrical rates model of character transitions denoted by pie charts at node positions. N=122. Abbreviations: BIA, Biarmosuchia. CYN, Cynodontia. DIN, Dinocephalia. DSS, Deep shearing specialists. EOT, Eothyrididae. FFsG, Feeding functional subgroup. FG, Forceful grippers. GG, Gracile grippers. GRA, Grip and rip attackers. GRG, Gorgonopsia, OPH, Ophiacodontidae. PBS, Power bite specialists. SBS, Shearing bite specialists. SPH, Sphenacodontia (non-therapsid). RLA, Rapid light attacker. THR, Therocephalia. VAR, Varanopidae. Biarmosuchia, Dinocephalia and Therocephalia silhouettes by Dmitry Bogdanov (vectorized by T. Michael Keesey); All other silhouettes created by S.A.S., but some are vectorised from artwork by Felipe Alves Elias (https://www.paleozoobr.com/), available for academic use with attribution.

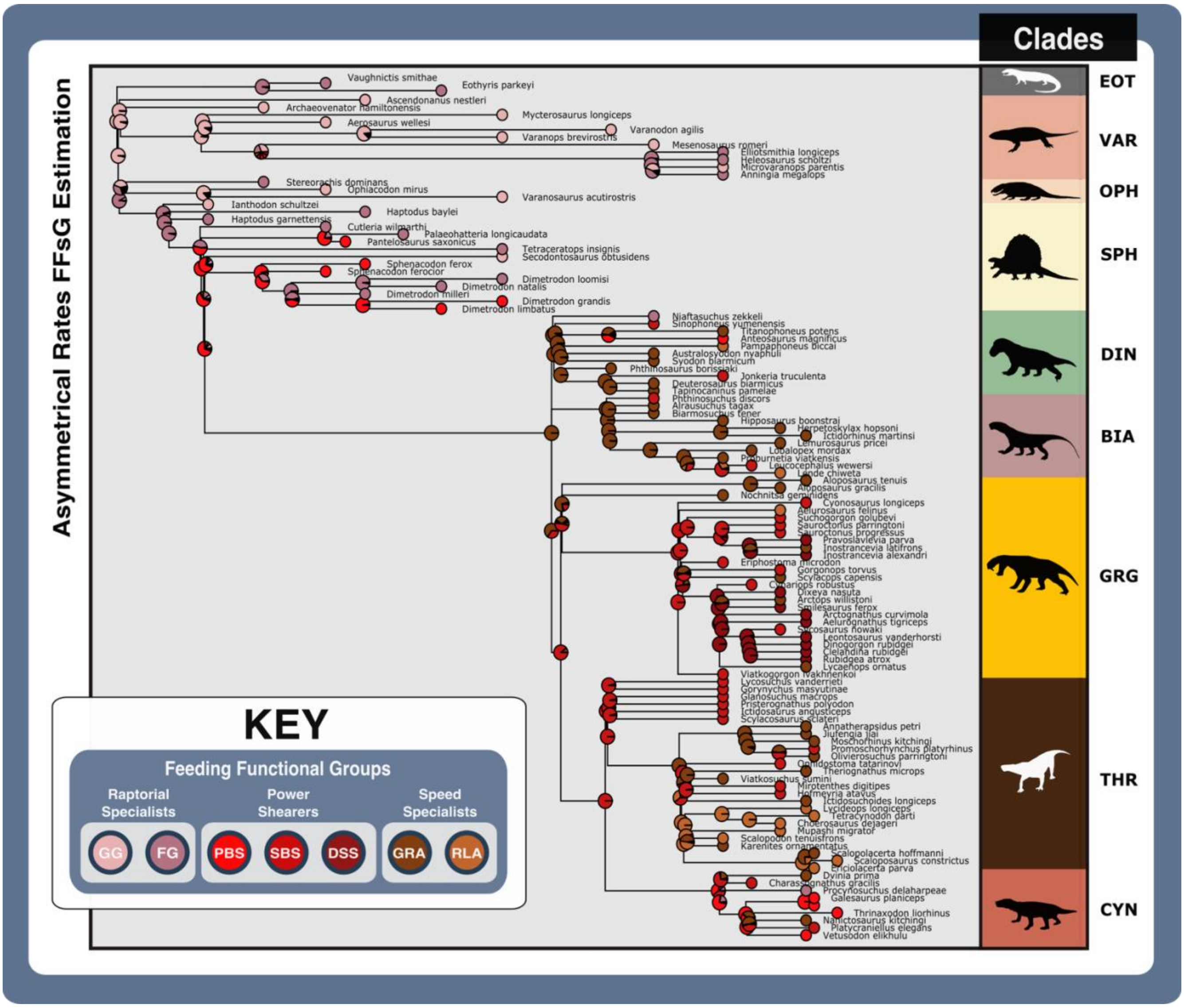

Supplementary Figure 11. Asymmetrical rates ancestral trait reconstruction of carnivorous synapsid FFsG through the late Palaeozoic. Feeding functional subgroup states cross the carnivorous synapsid phylogeny with reconstructed ancestral character state likelihoods under an asymmetrical rates model of character transitions denoted by pie charts at node positions. N=122. Abbreviations: BIA, Biarmosuchia. CYN, Cynodontia. DIN, Dinocephalia. DSS, Deep shearing specialists. EOT, Eothyrididae. FFsG, Feeding functional subgroup. FG, Forceful grippers. GG, Gracile grippers. GRA, Grip and rip attackers. GRG, Gorgonopsia, OPH, Ophiacodontidae. PBS, Power bite specialists. SBS, Shearing bite specialists. SPH, Sphenacodontia (non-therapsid). RLA, Rapid light attacker. THR, Therocephalia. VAR, Varanopidae. Biarmosuchia, Dinocephalia and Therocephalia silhouettes by Dmitry Bogdanov (vectorized by T. Michael Keesey); All other silhouettes created by S.A.S., but some are vectorised from artwork by Felipe Alves Elias (https://www.paleozoobr.com/), available for academic use with attribution.

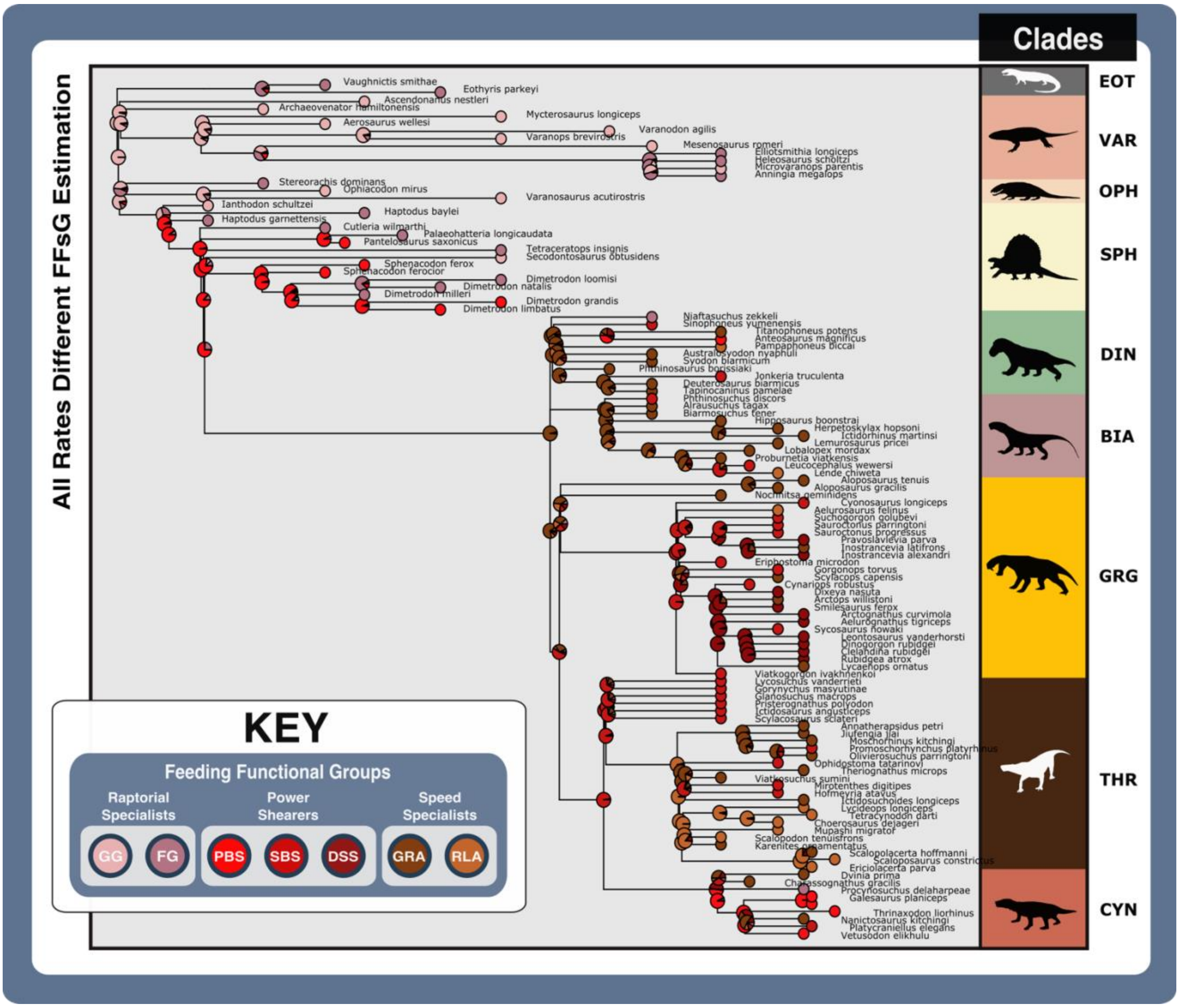

Supplementary Figure 12. All rates different ancestral trait reconstruction of carnivorous synapsid FFsG through the late Palaeozoic. Feeding functional subgroup states cross the carnivorous synapsid phylogeny with reconstructed ancestral character state likelihoods under an all rates different model of character transitions denoted by pie charts at node positions. N=122. Abbreviations: BIA, Biarmosuchia. CYN, Cynodontia. DIN, Dinocephalia. DSS, Deep shearing specialists. EOT, Eothyrididae. FFsG, Feeding functional subgroup. FG, Forceful grippers. GG, Gracile grippers. GRA, Grip and rip attackers. GRG, Gorgonopsia, OPH, Ophiacodontidae. PBS, Power bite specialists. SBS, Shearing bite specialists. SPH, Sphenacodontia (non-therapsid). RLA, Rapid light attacker. THR, Therocephalia. VAR, Varanopidae. Biarmosuchia, Dinocephalia and Therocephalia silhouettes by Dmitry Bogdanov (vectorized by T. Michael Keesey); All other silhouettes created by S.A.S., but some are vectorised from artwork by Felipe Alves Elias (https://www.paleozoobr.com/), available for academic use with attribution.

Supplementary Note 1

R Code

1. **Principle Component Analyses.**

```
#########Shape Data – landmarks#########
library(geomorph)

###Read in Data
TPPr_GML<-read.csv(file = "PermPred_01_2021_GML.csv", header=T, row.names=1)
TPPr_GML1<-arrayspecs(TPPr_GML, 59, 2)

### Plot all specimens
plotAllSpecimens(TPPr_GML1, mean = TRUE, links = NULL,)

### Run PCA
PCA<-gm.prcomp(TPPr_GML1)
plot(PCA, pch=16, labels=T)

### Get PCA results
summary(PCA)
Pcscore.res<-PCA$x  ### pc scores for each taxon

#########Functional Data#########
library(FactoMineR)

###Read in Data
TPermPr_master<-read.csv("TPermPr_data_01_2021_alt.csv", row.names=1, header=T)
TPermPr_FuncM<-TPermPr_master[,7:14] #Isolate raw mandibular functional character data as
separate data object.

### Run PCA – includes scaling/z standardisation.
TPermPr_FD.pca<-PCA(TPermPr_FuncM, scale.unit=T, ncp=10)

### Get PCA results
summary(TPermPr_FD.pca)
TPermPr_FD.pca$eig #Return eigenvalues.
TPermPr_FD.pca$ind$coord #Return PC scores.

dimdesc(TPermPr_FD.pca) #Visually diplays the character composition of PC 1 & 2.
TPermPr.pca.loadings<-
sweep(TPermPr_FD.pca$var$coord,2,sqrt(TPermPr_FD.pca$eig[1:ncol(TPermPr_FD.pca$var$coord
),1]),FUN="/") #Return PCA loadings.
TPermPr.pca.loadings

TPermPr_FmPC<-data.matrix(TPermPr_FD.pca$ind$coord) #Create PC scores object
```

2. **Cluster Analyses.**

```
library(factoextra)

###Read in Data
TPermPr_master<-read.csv("TPermPr_data_01_2021_alt.csv", row.names=1, header=T)
TPermPr_FuncM<-TPermPr_master[,7:14] #Isolate raw mandibular functional character data as
separate data object.
```

TPermPr_FFsG_Zsc<-scale(TPermPr_FuncM, center=T, scale=T) #Z scale & center data.

Run Exploratory Hierarchical Cluster Analysis.
FMres.hc <- eclust(TPermPr_Fm_Zsc, FUNcluster = "hclust", k.max=10, hc_metric="euclidean", hc_method="ward.D2", nboot=4000).
View initial results.
FMres.hc
FMres.hc_$cluster #View cluster assignments for each taxon
plot(FMres.hc, cex = 0.5)

Run Hierarchical Cluster Analysis.
Use above results to specify range of cluster numbers to consider classifying taxa into.
FMres.hc_RV <- eclust(TPermPr_Fm_Zsc, FUNcluster = "hclust", k.max=2,3,4,5,6,7, hc_metric="euclidean", hc_method="ward.D2", nboot=4000)
View results.
FMres.hc_RV
FMres.hc_RV$cluster #View cluster assignments for each taxon
plot(FMres.hc_RV, cex = 0.5)

Run Partition Cluster Methods
#K-means Analysis #for PAM, in FUNcluster replace “kmeans” with “pam”
##Cluster range set as used in analysis for all taxa.
FMres.Km<-eclust(TPermPr_Fm_Zsc, FUNcluster = "kmeans", k.max=2,3,4,5,6,7, nstart=200, nboot=1000) #Should automatically plot result in R plot window.

View results.
FMres.Km
FMres.Km$cluster #View cluster assignments for each taxon

3. **Disparity Analyses.**
library(dispRity)

###Read in Data
PhyloAL_Data<-read.csv("TPermPred_TreeALigned_Data.csv", header = T, row.names = 1)
PhyloAL_Data1<-as.matrix(PhyloAL_Data)

###Read in time-scaled phylogeny
PPred.tree<-readRDS("Perm_Pred_time-scaled_tree.rds") #read in tree R file.
Chronotree2a<-PPred.tree

Subset First and Last Appearance Dates (FADs & LADs).
fADlAD=as.data.frame(PhyloAL_Data1[,128:129])

Set Timebins to be compared
timesbinAge<-c(307.0, 303.7, 298.9, 295, 290.1, 283.5, 272.95, 268.8, 265.10, 259.1, 254.14, 251.9, 251.2) #Palaeozoic

###Following example uses Shape PC scores – substitute fPCdata for PCdata to run disparity analyses with functional data.
#PCdata<- PhyloAL_Data1 [,1:118] #Shape PC scores
#fPCdata<- PhyloAL_Data1 [,119:125] #Functional PC scores

###Phylogenetic Disparity Time Series Analysis (Sum of Variance)

```
TPermPred_disparity_data_gmPC<-dispRity(boot.matrix(chrono.subsets(PhyloAL_Data1[,1:118], PPred.tree, time = timesbinAge, model="proximity", method = "c", FADLAD=fADlAD), bootstraps = 1000, rarefaction = 2), metric = c(sum, variances))

TPermPred_disparity_data_PC<-TPermPred_disparity_data_gmPC
plot(TPermPred_disparity_data_PC, type = "continuous")

#Create disparity results object
TPermPred.SOV.final <- summary(TPermPred_disparity_data_PC, quantiles = c(50,90,95), cent.tend = mean, digits = 10)
TPermPred.metric.plot <- TPermPred.SOV.final
TPermPred.metric.plot
TPermPred.plot.data <-
c(as.integer(rownames(TPermPred.metric.plot[match(unique(TPermPred.metric.plot$subset), TPermPred.metric.plot$subset),])))
TPermPred.plot.data
TPermPred.main.res <- TPermPred.metric.plot[, "bs.mean"][TPermPred.plot.data] # select rows required #mean value
TPermPred.lower <- TPermPred.metric.plot[, "2.5%"][TPermPred.plot.data] #Lower 95% CI
TPermPred.upper <- TPermPred.metric.plot[, "97.5%"][TPermPred.plot.data] #Upper 95% CI
TPermPred.disp.res<-cbind(TPermPred.main.res,TPermPred.lower,TPermPred.upper) #Create one object with results and upper and lower bounds.
TPermPred.disp.res

###Phylogenetic Disparity Macroevolutionary Modelling###.
TPermPred _model_disp_time <- model.test(data = TPermPred_disparity_data_PC, model = c("Stasis", "BM", "OU", "Trend", "EB"), pool.variance = NULL, fixed.optima = T)
TPermPred _model_disp_time

#Plot model results.
plot(TPermPred _model_disp_time, ylim=c(0,1))
```

4. **Phylogenetic Trait Estimations.**

```
###Run Phylogenetic Discrete Trait Estimations (Maximum Likelihood)###.
###Read in discrete FFsG data
TraitFile<-read.csv("ASR_characters.csv", row.names = 1)
tp<-TraitFile

######Equal Rates Model (ERM)
fitERM <- asr_mk_model(timetree_scaled, n_states, tip_states = NULL, tip_priors = tp, rate_model=”ER”, reroot = T, Ntrials=10)
#View model data
fitERM$transition_matrix
fitERM$loglikelihood
fitERM$AIC

######Symmetric Model (SYM)
fitSYM <- asr_mk_model(timetree_scaled, n_states, tip_states = NULL, tip_priors = tp, rate_model="SYM", reroot = T, Ntrials=10)

######All Rates Different Model (ARD)
fitARD <- asr_mk_model(timetree_scaled, n_states, tip_states = NULL, tip_priors = tp, rate_model="ARD", reroot = F, Ntrials=10)

######Asymmetric Model (ASYM)
```

```
asym <- matrix(0, n_states, n_states)
asym[lower.tri(asym)] <- 1
asym[upper.tri(asym)] <- 2
asym

fitASYM <- asr_mk_model(timetree_scaled, n_states, tip_states = NULL, tip_priors = tp,
rate_model=asym, reroot = F, Ntrials=10).

######Model Average
mdl_avg <- (fitER$ancestral_likelihoods * AIC_res[1, 6]) + (fitSYM$ancestral_likelihoods *
AIC_res[2, 6]) + (fitARD$ancestral_likelihoods * AIC_res[3, 6]) + (fitASYM$ancestral_likelihoods
* AIC_res[4, 6])
```

Supplementary References

1. Singh, S. A., Elsler, A., Stubbs, T. L., Bond, R., Rayfield, E. J., & Benton, M. J. Niche partitioning shaped herbivore macroevolution through the early Mesozoic. *Nat. Commun.* **12**, 1-13. (2021).
2. Westneat, M. W. Transmission of force and velocity in the feeding mechanisms of labrid fishes (Teleostei, Perciformes). *Zoomorphology*, **114,** 103-118 (1994).
3. Westneat, M. W. Evolution of levers and linkages in the feeding mechanisms of fishes. *Integr. Comp. Biol.* **44,** 378-389 (2004).
4. Wainwright, P. C., & Richard, B. A. Predicting patterns of prey use from morphology of fishes. *Environ. Biol. Fishes* **44**, 97-113 (1995).
5. Stubbs, T. L., Pierce, S. E., Rayfield, E. J., & Anderson, P. S. Morphological and biomechanical disparity of crocodile-line archosaurs following the end-Triassic. *Proc. R. Soc. B.* **280,** 20131940. (2013).
6. Kemp T. S. The origin and evolution of mammals. (Oxford Univ. Press. Oxford. 2005).
7. Crompton, A. W. The evolution of the mammalian jaw. *Evolution* **17**, 431-439. (1963).
8. DeMar, R., & Barghusen, H. R. Mechanics and the evolution of the synapsid jaw. *Evolution* **26**, 622-637. (1972).
9. Lautenschlager, S., Gill, P., Luo, Z. X., Fagan, M. J., & Rayfield, E. J. Morphological evolution of the mammalian jaw adductor complex. *Biol. Rev.* **92**, 1910-1940. (2017).
10. Angielczyk, K. D., & Kammerer, C. F. 5. Non-mammalian synapsids: the deep roots of the mammalian family tree. In *Handbook of zoology: mammalian evolution diversity and systematics* (eds Zachos, F. E., Asher, R. J.), 117-198. (Berlin, Germany: De Gruyter. 2018)
11. Barghusen, H. R. The adductor jaw musculature of Dimetrodon (Reptilia, Pelycosauria). *J. Paleontol.* **47**, 823-834. (1973).
12. MacLaren, J. A., Anderson, P. S., Barrett, P. M., & Rayfield, E. J. Herbivorous dinosaur jaw disparity and its relationship to extrinsic evolutionary drivers. *Paleobiology* **43**, 15-33 (2017).
13. Sakamoto, M. Jaw biomechanics and the evolution of biting performance in theropod dinosaurs. *Proc. R. Soc. B.*, **277**, 3327-3333. (2010).
14. Barghusen, H. R. The lower jaw of cynodonts (Reptilia, Therapsida) and the evolutionary origin of mammal-like adductor jaw musculature. *Postilla* **116**, 1-49. (1968).
15. Kemp, T. S. On the functional morphology of the gorgonopsid skull. *Philos. Trans. R. Soc.* **256**, 1-83. (1969).
16. Olroyd, S. L., & Sidor, C. A. Nomenclature, comparative anatomy, and evolution of the reflected lamina of the angular in non-mammalian synapsids. . *J. Vertebr. Paleontol*, **42**, e2101923. (2022).
17. Botha, J., Abdala, F., & Smith, R. The oldest cynodont: new clues on the origin and early diversification of the Cynodontia. *Zool. J. Linn. Soc.*, **149**, 477-492. (2007).

18. Anderson, P. S., & Westneat, M. W. Feeding mechanics and bite force modelling of the skull of Dunkleosteus terrelli, an ancient apex predator. *Biol. Lett.* **3**, 77-80 (2006).
19. Anderson, P. S., Friedman, M., Brazeau, M. D., & Rayfield, E. J. Initial radiation of jaws demonstrated stability despite faunal and environmental change. *Nature* **476**, 206–209 (2011).
20. MacLaren, J. A., Anderson, P. S., Barrett, P. M., & Rayfield, E. J. Hebivorous dinosaur jaw disparity and its relationship to extrinsic evolutionary drivers. *Paleobiology* **43**, 15-33 (2017).
21. Button, D. J., Rayfield, E. J., & Barrett, P. M. Cranial biomechanics underpins high sauropod diversity in resource-poor environments. *Proc. R. Soc. B Biol. Sci.* **281**, 20142114 (2014).
22. Daegling, D. J. Biomechanical scaling of the hominoid mandibular symphysis. *J. Morphol.,* **25**, 12-23 (2001).
23. Jones, M. E., O'higgins, P., Fagan, M. J., Evans, S. E., & Curtis, N. Shearing mechanics and the influence of a flexible symphysis during oral food processing in Sphenodon (Lepidosauria: Rhynchocephalia). *Anat. Rec.*, **295**, 1075-1091 (2012).
24. Porro, L. B., Holliday, C. M., Anapol, F., Ontiveros, L. C., Ontiveros, L. T., & Ross, C. F. Free body analysis, beam mechanics, and finite element modeling of the mandible of *Alligator mississippiensis*. *J. Morphol.*, **272**, 910-937 (2011).
25. Walmsley, C.W., et al. Why the long face? The mechanics of mandibular symphysis proportions in crocodiles. *PLoS One*, **8**, e53873 (2013).
26. Janis, C. Correlations between craniodental morphology and feeding behavior in ungulates: reciprocal illumination between living and fossil taxa. *Functional Morphology in Vertebrate Paleontology* (Eds Thomason J. J.), 76-98 (Cambridge Univ. Press, Cambridge, 1995).
27. Ramsay, J. B., & Wilga, C. D. Morphology and mechanics of the teeth and jaws of white-spotted bamboo sharks (*Chiloscyllium plagiosum*). *J. Morphol.* **268**, 664-682 (2007).
28. Sookias, R. B.; Butler, R. J. & Benson, R. B. J. Rise of dinosaurs reveals major body-size transitions are driven by passive processes of trait evolution. *Proc. R. Soc. B.* **279**, *2180-2187* (2012).
29. Mann, A. & Paterson, R. S**.** Cranial osteology and systematics of the enigmatic early `sail-backed' synapsid *Echinerpeton intermedium* Reisz, 1972, and a review of the earliest `pelycosaurs' *J. Syst. Palaeontol.* **18**, 529-539 (2020).
30. Benson, R. B. J. Interrelationships of basal synapsids: cranial and postcranial morphological partitions suggest different topologies. *J. Syst. Palaeontol.* **10**, 601–624. (2012).
31. Reisz, R. R. and Fröbisch, J. The oldest caseid synapsid from the Late Pennsylvanian of Kansas, and the evolution of herbivory in terrestrial vertebrates. *PLoS ONE* **9**, e94518. (2014).
32. Brocklehurst, N., Reisz, R. R., Fernandez, V., and Fröbisch, J. A re-description of ‘Mycterosaurus’ smithae, an Early Permian eothyridid, and its impact on the phylogeny of pelycosaurian-grade synapsids. *PLoS ONE* **11**, e0156810. (2016).

33. Brink, K. S., Maddin, H. C., Evans, D. C., and Reisz, R. R. Re-evaluation of the historic Canadian fossil Bathygnathus borealis from the Early Permian of Prince Edward Island. *Can. J. Earth Sci.* **52**, 1109–1120. (2015).
34. Liu, J. Osteology, ontogeny, and phylogenetic position of Sinophoneus yumenensis (Therapsida, Dinocephalia) from the Middle Permian Dashankou Fauna of China. *J. Vertebr. Paleontol.* **33**, 1394–1407. (2013).
35. Bendel, E. M., Kammerer, C. F., Kardjilov, N., Fernandez, V., & Fröbisch, J. Cranial anatomy of the gorgonopsian Cynariops robustus based on CT-reconstruction. *PloS one*, **13**, e0207367. (2018).
36. Liu, J. and Abdala, F. The tetrapod fauna of the upper Permian Naobaogou Formation of China: 3. Jiufengia jiai gen. et sp. nov., a large akidnognathid therocephalian. *PeerJ*, **7**, e6463. (2019).
37. Abdala, F., Gaetano, L. C., Smith, R. M. H., and Rubidge, B. S. A new large cynodont from the Late Permian (Lopingian) of the South African Karoo Basin and its phylogenetic significance. *Zool. J. Linn. Soc.* **186**, 983–1005. (2019).
