# Supplementary material for: Predatory synapsid ecomorphology signals growing dynamism of late Palaeozoic terrestrial ecosystems
Source: Commun Biol. 2024 Feb 17;7:201. doi: 10.1038/s42003-024-05879-2 (PMC10874460; doi:10.1038/s42003-024-05879-2)
Supplement: Supplementary file 3 — Description of Additional Supplementary Files [file 42003_2024_5879_MOESM3_ESM.pdf]

Description of Additional Supplementary Files

File name: Supplementary Data 1
Description: Procrustes-aligned jaw landmark data. (Source data for Figs. 1, 4-5).

File name: Supplementary Data 2
Description: Raw jaw functional measurement data. (Source data for Figs. 1-2, 6-7).

File name: Supplementary Data 3
Description: Body size data.

File name: Supplementary Data 4
Description: Functional feeding group (FFG) and subgroup classifications. (Source data for Figs. 2, 6-7.)

File name: Supplementary Data 5
Description: Age ranges.

File name: Supplementary Data 6
Description: Functional feeding group assignments for ancestral trait estimations. (Source data for Figs. 6 and 7).

File name: Supplementary Data 7
Description: Functional feeding groups in each timebin (Source data for Fig. 6).

File name: Supplementary Data 8
Description: Linear discriminant analysis FFG classifications. (Source data for Supplementary Fig. 6).

File name: Supplementary Data 9
Description: Functional feeding groups across assemblages through time. (Source data for Fig. 8).

File name: Supplementary Data 10
Description: Late Palaeozoic Assemblage Data (Source data for Figs. 6 and 8).

File name: Supplementary Data 11
Description: Jaw image source references.

File name: Supplementary Data 12
Description: Femur length data source references.
