# Supplementary material for: Predatory synapsid ecomorphology signals growing dynamism of late Palaeozoic terrestrial ecosystems
Source: Commun Biol. 2024 Feb 17;7:201. doi: 10.1038/s42003-024-05879-2 (PMC10874460; doi:10.1038/s42003-024-05879-2)
Supplement: Supplementary file 5 — Reporting Summary [file 42003_2024_5879_MOESM5_ESM.pdf]

Corresponding author(s): Suresh A. SINGH

Last updated by author(s): Jan 5, 2024

Reporting Summary

Nature Research wishes to improve the reproducibility of the work that we publish. This form provides structure for consistency and transparency in reporting. For further information on Nature Research policies, see Authors & Referees and the Editorial Policy Checklist.

Statistics

For all statistical analyses, confirm that the following items are present in the figure legend, table legend, main text, or Methods section.

n/a	Confirmed	
☐	☒	The exact sample size (n) for each experimental group/condition, given as a discrete number and unit of measurement
☐	☒	A statement on whether measurements were taken from distinct samples or whether the same sample was measured repeatedly
☐	☒	The statistical test(s) used AND whether they are one- or two-sided<br>*Only common tests should be described solely by name; describe more complex techniques in the Methods section.*
☐	☒	A description of all covariates tested
☐	☒	A description of any assumptions or corrections, such as tests of normality and adjustment for multiple comparisons
☐	☒	A full description of the statistical parameters including central tendency (e.g. means) or other basic estimates (e.g. regression coefficient) AND variation (e.g. standard deviation) or associated estimates of uncertainty (e.g. confidence intervals)
☐	☒	For null hypothesis testing, the test statistic (e.g. F, t, r) with confidence intervals, effect sizes, degrees of freedom and P value noted<br>*Give P values as exact values whenever suitable.*
☒	☐	For Bayesian analysis, information on the choice of priors and Markov chain Monte Carlo settings
☒	☐	For hierarchical and complex designs, identification of the appropriate level for tests and full reporting of outcomes
☒	☐	Estimates of effect sizes (e.g. Cohen's d, Pearson's r), indicating how they were calculated

Our web collection on statistics for biologists contains articles on many of the points above.

Software and code

Policy information about availability of computer code

Data collection	TPSUtil (version 1.58), TPSDig2 (version 2.17), and TPSrelw (version 1.61) were used to generate landmark morphometric data. ImageJ (version 1.52) was used to measure functional character data.
Data analysis	Principal component analyses were executed in R (version 3.6.1) using the 'geomorph' and 'FactoMineR' r packages to process the shape and functional data respectively. Cluster analyses were also performed in R using the 'FactoExtra' r package. Phylogenetic disparity through time and macroevolutionary modelling was implemented using the 'DispRity' r package. Ancestral trait reconstruction was carried out using the 'castor' and 'phytools' R packages for discrete and continuous traits, respectively. R code to run the analyses has been included in the Supplementary Information under the Supplementary Code section. One-way NPMANOVA tests were performed in PAST (version 3.24).

For manuscripts utilizing custom algorithms or software that are central to the research but not yet described in published literature, software must be made available to editors/reviewers. We strongly encourage code deposition in a community repository (e.g. GitHub). See the Nature Research guidelines for submitting code & software for further information.

Data

Policy information about availability of data

All manuscripts must include a data availability statement. This statement should provide the following information, where applicable:

- Accession codes, unique identifiers, or web links for publicly available datasets
- A list of figures that have associated raw data
- A description of any restrictions on data availability

Data are provided with the manuscript are lodged with Dryad at https://doi.org/10.5061/dryad.jq2bvq8h0.

Field-specific reporting

Please select the one below that is the best fit for your research. If you are not sure, read the appropriate sections before making your selection.

☐ Life sciences ☐ Behavioural & social sciences ☒ Ecological, evolutionary & environmental sciences

For a reference copy of the document with all sections, see nature.com/documents/nr-reporting-summary-flat.pdf

Ecological, evolutionary & environmental sciences study design

All studies must disclose on these points even when the disclosure is negative.

Study description	A comparative study of carnivorous synapsids through the late Palaeozoic using quantitative geometric and linear morphometric methods. Using mandible anatomy and body size, which are closely linked to trophic ecology, we infer distinct or common feeding functionality to identify evolutionary patterns of terrestrial carnivore evolution and the potential drivers of their evolution. Furthermore, by tracking carnivore diversification, we hope to track the wider diversification of terrestrial tetrapod faunas through this interval.
Research sample	Our sample includes all valid synapsid taxa from clades previously identified in the literature as carnivorous, through the Late Carboniferous to Early Triassic, with sufficiently complete mandibular material.
Sampling strategy	Sampling was conducted using a list of all valid carnivorous synapsid taxa from the Late Carboniferous to Early Triassic, using a published dataset from (Benton et al. 2013, Palaeogeogr. Palaeoclimatol. Palaeoecol.) This was updated using the latest literature to incorporate new taxa and taxonomic revisions. From this initial list of suitable taxa, we tried to source as many suitable mandible images as possible from the literature.
Data collection	Suresh A. Singh collected all the images used to generate mandibular morphometric data from the literature. Armin Elsler collected the femur length data, and the stratigraphic ranges for all taxa from the literature.
Timing and spatial scale	Late Carboniferous to Early Triassic (315.2- 251.2 million years ago).
Data exclusions	We excluded taxa that lacked sufficiently complete mandibular material. We also excluded the shape (landmark) data from the cluster analyses as this data was believed to be subject to additional (phylogenetic) influence and so an unclear indicator of mandible eco-functionality.
Reproducibility	Analyses re-run in R and PAST to ensure the reproducibility of results.
Randomization	N/A
Blinding	N/A

Did the study involve field work? ☐ Yes ☒ No

Reporting for specific materials, systems and methods

We require information from authors about some types of materials, experimental systems and methods used in many studies. Here, indicate whether each material, system or method listed is relevant to your study. If you are not sure if a list item applies to your research, read the appropriate section before selecting a response.

Materials & experimental systems

n/a	Involved in the study	
☒	☐	Antibodies
☒	☐	Eukaryotic cell lines
☐	☐	Palaeontology
☒	☐	Animals and other organisms
☒	☐	Human research participants
☒	☐	Clinical data

Methods

n/a	Involved in the study	
☒	☐	ChIP-seq
☒	☐	Flow cytometry
☒	☐	MRI-based neuroimaging

Palaeontology

Specimen provenance	A full list of specimens used in this study is available within the Supplementary Data within S8 and S9. All specimens are from museum specimens, having been published in previous literature or photographed directly within museum collections.
Specimen deposition	All specimens are accessible to other researchers through published literature or as specimens within public museums.

Dating methods

Taxon ranges were collected from the literature and details are given within the methods and Supplementary Data (S4, S8, and S9).

☒ Tick this box to confirm that the raw and calibrated dates are available in the paper or in Supplementary Information.
